# YbeY is required for ribosome small subunit assembly and tRNA processing in human mitochondria

**DOI:** 10.1093/nar/gkab404

**Published:** 2021-05-25

**Authors:** Aaron R D’Souza, Lindsey Van Haute, Christopher A Powell, Christian D Mutti, Petra Páleníková, Pedro Rebelo-Guiomar, Joanna Rorbach, Michal Minczuk

**Affiliations:** Medical Research Council Mitochondrial Biology Unit, University of Cambridge, Hills Road, Cambridge CB2 0XY, UK; Medical Research Council Mitochondrial Biology Unit, University of Cambridge, Hills Road, Cambridge CB2 0XY, UK; Medical Research Council Mitochondrial Biology Unit, University of Cambridge, Hills Road, Cambridge CB2 0XY, UK; Medical Research Council Mitochondrial Biology Unit, University of Cambridge, Hills Road, Cambridge CB2 0XY, UK; Medical Research Council Mitochondrial Biology Unit, University of Cambridge, Hills Road, Cambridge CB2 0XY, UK; Medical Research Council Mitochondrial Biology Unit, University of Cambridge, Hills Road, Cambridge CB2 0XY, UK; Medical Research Council Mitochondrial Biology Unit, University of Cambridge, Hills Road, Cambridge CB2 0XY, UK; Medical Research Council Mitochondrial Biology Unit, University of Cambridge, Hills Road, Cambridge CB2 0XY, UK

## Abstract

Mitochondria contain their own translation apparatus which enables them to produce the polypeptides encoded in their genome. The mitochondrially-encoded RNA components of the mitochondrial ribosome require various post-transcriptional processing steps. Additional protein factors are required to facilitate the biogenesis of the functional mitoribosome. We have characterized a mitochondrially-localized protein, YbeY, which interacts with the assembling mitoribosome through the small subunit. Loss of YbeY leads to a severe reduction in mitochondrial translation and a loss of cell viability, associated with less accurate mitochondrial tRNA^Ser(AGY)^ processing from the primary transcript and a defect in the maturation of the mitoribosomal small subunit. Our results suggest that YbeY performs a dual, likely independent, function in mitochondria being involved in precursor RNA processing and mitoribosome biogenesis. Issue Section: Nucleic Acid Enzymes.

## Graphical Abstract

**Graphical Abstract:**
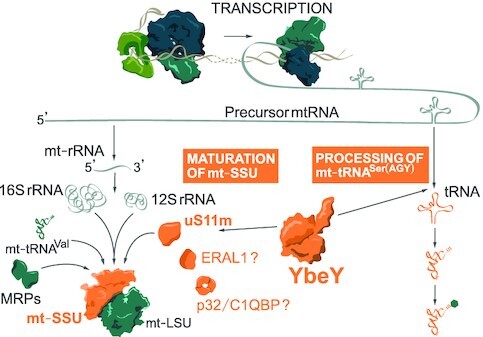
YbeY is involved in maturation of the mt-SSU and processing of precursor RNA.

## INTRODUCTION

Oxidative phosphorylation (OxPhos) is one of the major functions of mitochondria. A majority of the components of the OxPhos complexes are encoded in the cell nucleus and imported into mitochondria upon translation in the cytoplasm. However, thirteen structural polypeptides of the OxPhos complexes are translated within mitochondria. These polypeptides are encoded in the human mitochondrial genome (mtDNA) along with the 22 tRNAs and two rRNAs that make up the RNA components of the intra-organellar translation apparatus.

The mtDNA is transcribed bidirectionally, generating long polycistronic RNA strands. In the majority of cases, mRNAs and rRNAs in the precursors are flanked by tRNA molecules (1,2). The excision of these mt-tRNAs is performed by endonucleolytic enzymes RNase P at the 5′ end, and RNase Z (ELAC2) at the 3′ end (3,4). This releases the mt-mRNAs and mt-rRNAs, with the latter undergoing further maturation and assembly into the mitochondrial ribosome.

The mammalian mitoribosome consists of a large (mt-LSU) and small subunit (mt-SSU), and is composed of 82 nuclear-encoded proteins, two mt-rRNAs and one mt-tRNA(5,6). The assembly of the mitoribosome involves the chemical modification of the mt-rRNAs, spontaneous RNA–protein and RNA–RNA interactions and RNA folding, and requires the assistance of various assembly factors. Several enzymes introducing post-transcriptional modifications to mt-rRNA have been recently shown to play a role in mitoribosome assembly (7–9). The assembly factors include, but are not limited to, GTPases and ATP-dependent RNA helicases. The hydrolysis of NTPs by these enzymes ensure that the mt-rRNA does not fall into conformational traps. For example, GTPBP5 (also called OBGH1) (10,11), GTPBP6 (12), GTPBP7 (MTG1) (13) and GTPBP10 (also called ObgH2) (14,15) are four conserved GTPases that assist in the assembly of mt-LSU. GTPBP5 and GTPBP10 functionally interact with other late-stage assembly factors including NSUN4, MTERF4 and MRM2 (16,17). C4orf14 (18–20) and ERAL1 (21,22) are two conserved GTPases that are involved with the biogenesis of mt-SSU. During mitoribosome assembly, RNA helicases such as DDX28 (23) and DHX30 (24) unwind mt-rRNA coupled to the hydrolysis of ATP. Additional factors such as MALSU1 form a trimeric complex with L0R8F8 and mt-ACP at the intersubunit interface preventing the association of the immature subunits during monosome formation (25–27). The early stages of mitoribosomal assembly occur co-transcriptionally in the nucleoid (28). The assembly of mt-LSU can initiate on the 12S–16S mt-rRNA precursor molecule, but not the mt-SSU (29). The initiation and progression of small subunit assembly may be inhibited until a certain stage of early mt-LSU assembly is complete (15,30). Then, subsequent steps of mitoribosome biogenesis are continued in distinct foci called RNA granules (31,32).

In bacteria, the ribosomal RNAs are co-transcribed as a single operon. The pre-rRNA undergoes the removal of transcribed spacer regions before the rRNA is ready for assembly. The bacterial homolog of human YbeY is a single strand-specific endoribonuclease that is responsible for the 3′ end processing of the SSU rRNA (33–36). Depletion of the protein leads to the accumulation of precursors, the loss of the SSU and a subsequent translation defect (37,38). In addition, the protein also plays a role in LSU rRNA and 5S rRNA maturation (34). However, recent work suggests that, unlike other nucleases that can use immature small subunits containing precursor 16S rRNA as substrates for nucleolytic activity, the endonucleolytic activity of purified bacterial YbeY on precursor 16S SSU rRNA has not been demonstrated *in vitro*. Even the addition of various combinations of known YbeY interactors, ribosome components and initiator tRNA did not lead to expected YbeY-specific RNA cleavage events (35,39). Furthermore, it has also been implicated in the degradation of defective and misprocessed SSU rRNA and, as such, plays a role in ribosome quality control, in conjunction with RNase R (34,39,40).

Little is known about the human orthologue of bacterial YbeY. Unlike bacteria, the 16S and 12S mt-rRNA components are flanked by mt-tRNAs, and their excision by RNase P and ELAC2, without the need for further end processing, is sufficient for assembly into the mitochondrial ribosome (41). Therefore, the function of mammalian YbeY is expected to be substantially different. Recent work demonstrated that the introduction of human YbeY into bacteria lacking the homolog caused partial rescue of phenotype (42). However, the exact function of YbeY in mammalian mitochondria has so far not been investigated in details.

We and others have recently shown that defects in the structural components of mitoribosome and its assembly factors can lead to a human disorder of mitochondrial respiration (43–46). The RNase activity of YbeY from various bacterial species (34,36,47), plant chloroplasts (48) and the mammalian mitochondria have been previously demonstrated (42). Therefore, we have focussed on the functional significance of the protein in the mitochondria. Here, we show that YbeY is a mitochondrially-localized protein. We characterize YbeY-deficient HEK293T cell lines and YbeY knockout Hap1 cell lines. We demonstrate that the loss of the protein leads to a severe perturbation of mitochondrial translation. We also show that YbeY has a dual function: (i) YbeY plays a role in tRNA^Ser(AGY)^ processing at both the 5′ and 3′ ends and (ii) interacts with the mt-SSU component uS11m. In certain growth conditions, the loss of YbeY causes a decrease in the abundance of 12S mt-rRNA and mt-SSU. These findings on the role of YbeY provide new insights into the mitoribosome assembly process and, in the long-term, can help pave the way for the development of future mechanism-based therapies for mitochondrial diseases.

## MATERIALS AND METHODS

### Plasmids

The plasmid encoding the open reading frame (ORF) of YbeY was purchased as an IMAGE cDNA clone (IRAUp969G02109D; GeneScript Item number: SC1010) from Source Bioscience. The YbeY ORF was equipped with the FLAG-tag coding sequence (encoded on the reverse PCR primer) and cloned into the pcDNA5/FRT/TO plasmid or an untagged version was cloned into pcDNA5/FRT/TO without or with the FLAG/Strep2 tag, as described in (49). The p32/C1QBP ORF (a kind gift of Dr Jiuya He, MRC-MBU) was cloned into pcDNA5/FRT/TO harbouring FLAG/Strep2 tag upon PCR amplification. The primers used are detailed in Supplementary Table S1.

### Genome-modified cell lines

For the generation of YbeY deficient HEK293T cell lines, the cells were transiently transfected with CompoZr ZFNs (Sigma-Aldrich) using Cell Line Nucleofector (Lonza), buffer kit V (Lonza) and program A-023. Seventy-two hours after transfection, the cells were single-cloned into 96 well plates and screened using Sanger sequencing to identify indels at the ZFN target site and western blotting. Wild-type Hap1 and YbeY knockout Hap1 cell lines were purchased from Horizon Discovery (Product ID: HZGHC002765c022).

### Cell culture

Cell lines were maintained at 5% CO_2_ and 37°C in humidified incubators. The HEK293T and HeLa cells were cultured in Dulbecco's Modified Eagle Medium (DMEM), containing 4.5 g/l glucose, 110 mg/l sodium pyruvate, supplemented with 10% (v/v) foetal bovine serum, 100 U/ml penicillin and 100 μg/ml streptomycin. Hap1 cells were cultured in Iscove's modified Dulbecco's medium (IMDM) containing 4.5 g/l glucose, 110 mg/l sodium pyruvate, supplemented with 10% (v/v) foetal bovine serum, 100 U/ml penicillin and 100 μg/ml streptomycin. IMDM for YbeY knockout Hap1 cells were supplemented with 20% FBS. HEK293T/Flp-In/T-Rex cells were cultured in supplemented DMEM with additional 15 μg/ml blasticidin and 100 μg/ml Zeocin. After transfection with pcDNA/FRT/TO plasmid, the cells were cultured in supplemented DMEM containing 4.5 g/l glucose, 110 mg/l sodium pyruvate, supplemented with 10% (v/v) tetracycline-free foetal bovine serum, 100 U/ml penicillin and 100 μg/ml streptomycin, 15 μg/ml blasticidin and 50 μg/ml hygromycin.

### Transfection

2 μg of DNA and 3 μl of Lipofectamine 2000 were incubated in 100 μl of OptiMEM separately for 5 minutes at room temperature. They were mixed and incubated for a further 10 min. Then, they were added to cells in supplemented DMEM and mixed by swirling.

### Immunodetection of proteins

A confluent 9 cm dish of HEK293T or Hap1 cells were washed with PBS and pelleted at 270 × g for 3 min. The pellet was lysed with Lysis buffer (50 mM Tris–HCl, 150 mM NaCl, 1 mM EDTA, 1% Triton, 1× Roche inhibitor tablet) on ice for 15 minutes and centrifuged at 6800 × g for 5 min. The protein concentration of the supernatant was quantified. 25 μg of lysate was boiled at 95°C with 33.3% NuPAGE LDS 4× sample buffer containing 200 mM DTT. The boiled solution was run on a 4–12% Bis–Tris NuPage polyacrylamide gel at 200 V for 30 min. The proteins were transferred onto a nitrocellulose membrane with the iBlot 2 Dry Blotting System using Protocol ‘P0’. Gels were stained with SimplyBlue Safestain. The membrane was blocked with 5% non-fat milk in PBS-T (Phosphate-Buffered Saline, 0.1% Tween 20, pH 7.4) at room temperature. The membrane was then incubated overnight at 4°C with primary antibody diluted in 5% milk in PBS-T. The membrane was washed 3 times for 10 min with PBS-T. The membrane was incubated with secondary antibody diluted in PBS-T and then washed again as before. Finally, the membrane was developed using ECL and X-ray film. The antibodies used for this analysis and their dilutions can be found in Supplementary Table S2.

### Mitochondrial Isolation and cell fractionation

Mitochondria were isolated using a cell homogenizer (isobiotec) as described by (50). For cell fractionation, the mitochondrial isolation protocol that uses a Dounce homogenizer adapted from (49) was used. Briefly, 15 confluent 15 cm dishes of HEK293T cells were washed with 1× PBS and pelleted. The cell pellet was resuspended in 15 ml of mitobuffer (0.6 M mannitol, 10 mM Tris–HCl pH 7.4, 1 mM EDTA) containing 0.1% BSA. 100 μl of the cell suspension was kept aside as the ‘total fraction’. The suspension was homogenized in a Dounce homogenizer (15 strokes) and centrifuged at 400 × g for 10 min. The supernatant from this step was kept aside on ice. The pellet was resuspended in mitobuffer containing BSA and re-homogenized as above and centrifuged at 400 × g for 5 min. The pellet from this step was used as the ‘Debris fraction’. The supernatant from this step and the preceding step was centrifuged at 11 000 × g. The supernatant from this step was kept aside as the ‘Cytoplasm fraction’. The pellet was resuspended in 1 ml of mitobuffer containing 50 U/ml Benzonase, incubated on ice for 15 min and centrifuged at 11 000 × g for 10 min. The mitochondrial pellet was resuspended in 120 μl of mitobuffer and divided into two aliquots. The first aliquot was the mitochondrial fraction. The second aliquot was incubated with 4 μg/mg of Proteinase K for 30 min on ice (proteinase K fraction). The aliquots were centrifuged at 11 000 × g for 5 min. The pellet was lysed, and all fractions were analysed by western blotting.

### Immunocytochemistry

The cells were grown on coverslips in a well of a six-well plate. Twenty-four hours after transfection, the cells were washed with PBS and then incubated with 4% formaldehyde diluted in PBS for 15 min. The cells were then washed with PBS and then permeabilized for 5 min with 0.1% Triton-X diluted in PBS. The cells were incubated for 1 h in PBSS (5% FBS v/v, 95% PBS) and then for 1.5 h in primary antibody diluted in PBSS. The cells were washed three times for 5 min in PBSS. Secondary antibody, diluted in PBSS, was added to the cells and incubated for 1 h. Following the incubation, the cells were washed (5 min each) twice in PBSS and once in PBS and mounted in Vectashield mounting medium. The cells were visualized by fluorescence microscopy using a Zeiss LSM 510 META confocal microscope. The antibodies used for the immunocytochemistry analysis and their dilutions can be found in Supplementary Table S2.

### Circularization RT-PCR sequencing

For the analysis of the 5′ and 3′ ends of certain mt-tRNAs and mt-rRNAs, the protocol was adapted from (51). In brief, the RNA was circularized and reverse transcribed across the ligated junction. PCR was used to amplify the product, which was then cloned using a Zero Blunt TOPO PCR cloning kit and sequenced using an M13 forward universal sequencing primer. The primers for the circularization RT-PCR are listed in Supplementary Table S1.

### ^35^S-Methionine metabolic labelling of *de novo* protein synthesis

Newly synthesized mitochondrial proteins in Hap1 and HEK293T cell lines were labelled with ^35^S-labelled methionine and analysed by autoradiography as described by (16).

### Immunoaffinity purification and SILAC analysis

For SILAC-based immunoaffinity purification experiments, doxycycline-inducible YbeY-FLAG-expressing HEK293T cells were grown in SILAC DMEM media (ThermoFisher Scientific) containing heavy ^15^N- and ^13^C-labelled l-arginine (88 μg/ml) and l-lysine (152 μg/ml) or light ^14^N and ^12^C l-arginine (69 μg/ml) and l-lysine (146 μg/ml) (Sigma Aldrich). This medium also contained unlabelled l-proline (200 μg/ml). Cells were differentially induced with 100 ng/ml doxycycline for 24 h. The four conditions: heavy induced (HI), heavy uninduced (HU), light induced (LI), light uninduced (LU) were harvested, pelleted at 270 × g for 3 min, washed with PBS and re-pelleted. Uninduced cells were used as control. The cells were quantified and mixed in equal proportion as follows: HI with LU (control) and LI with HU (control). Mitochondria were isolated (see Mitochondrial isolation section) from the cell suspension. Mitochondria were lysed and immunoaffinity purification was carried out with FLAG M2 affinity gel (Sigma-Aldrich) following manufacturer's instructions. Elution was performed with FLAG peptide. The samples were analysed with LC–MS/MS on a Thermo Scientific Q Exactive Plus mass spectrometer and Proxeon EASY-nLC 1000 chromatography. The internal ‘lock mass’ standard of 445.120 *m*/*z* was used for accurate mass measurements. Proteins were identified using the ANDROMEDA algorithm of the MAXQUANT software package by comparing the proteins to the human UniProt database. The peptide information from both sets were combined and filtered using the PERSEUS software, removing proteins only identified by site, that matched a decoy peptide database and contaminating peptides. Proteins identified by two or fewer peptides were removed. Ratios for both reciprocal samples, i.e. HI/LU and LI/HU and a base two logarithm (log_2_) were calculated. The data was plotted on an X–Y scatter plot. The positive values for both log_2_ ratios (protein ID present in the top right quarter of the X-Y scatter plot) indicate interaction in the reciprocal experiments.

For non-SILAC immunoprecipitation experiments, the unlabelled induced cell expressing YbeY-FLAG/Strep2 were subjected to mitochondrial isolation and FLAG immunoaffinity purification, as described above. The samples were run on SDS-PAGE, the gel was stained for 1 h with SimplyBlue Safestain (ThermoFisher Scientific) and destained for 1 h using 20% methanol and 10% acetic acid solution. Visually identifiable bands were excised and submitted for analysis by mass spectrometry as described previously (49).

### Quantitative density gradient analysis by mass spectrometry (qDGMS) and complexome profiling analysis (ComPrAn)

In order to study mitoribosome composition and assembly in YbeY-KO Hap1 cells we used quantitative density gradient analysis by mass spectrometry (qDGMS) generally as described in (52). In this method, complexes are separated in near-native form by density gradient ultracentrifugation and SILAC enables simultaneous quantitative proteomic analysis of two biological samples. Samples were analysed with LC–MS/MS on a Thermo Scientific Oribitrap LTQ XL spectrometer with a nanospray inlet interface coupled with Proxeon Easy II nLC chromatography. Peptides were identified and quantified from raw data in Proteome Discoverer (Thermo Scientific). We further used the ComPrAn (complexome profiling analysis) R package to analyse and visualize the qDGMS datasets. The general approach to protein abundance estimation, normalization and data presentation in ComPrAn is based on the principles routinely used for the analysis of published SILAC complexome profiling experiments (53–55). The only difference from the published qDGMS protocol (52), was in-gel trypsin digestion, rather than digestion upon sample precipitation. Briefly, 100 μl sucrose gradient fractions were taken and run approximately 4 mm into a 4–12% Bis–Tris polyacrylamide gel to remove the sucrose. The gel was stained for 1 hour with SimplyBlue Safestain and destained for 1 hour (20% methanol, 10% acetic acid). The stained 4 mm gel region was cut and peptides from these gel slices were analysed by mass spectrometry following trypsin digestion as described in (52). The proteomics data are be available through CEDAR (www3.cmbi.umcn.nl/cedar/), an openly accessible database for depositing and exploring mass spectrometry data from complexome profiling studies. Compatibility and reusability of the data is ensured by a standardized reporting format containing the ‘minimum information required for a complexome profiling experiment’ (MIACE).

### RNA isolation, northern blotting and RT-qPCR

RNA was extracted from HEK293T and Hap1 cell lines with TRIzol reagent (ThermoFisher Scientific) according to manufacturer's instructions. Northern blot was performed as described by (49) with minor modifications. Total RNA was resolved on a 6% or a 15% urea–PAGE gel (for mt-tRNAs). After wet transfer of the RNA to a nylon membrane (Magnoprobe, 0.45 μ; GE Healthcare or Genescreenplus, NEN DuPont) in 2 x SSC (150 nM NaCl, 15 mM sodium citrate pH 7.0), the membrane was subjected to UV-crosslinking (0.12 J). High resolution tRNA gels were dry blotted using the ‘rapid transfer method’ where a dry nylon membrane is placed directly on the gel for 3 minutes and the RNA is UV-crosslinked to the membrane. The membranes were hybridized overnight at 65°C with radioactively labelled PCR fragments corresponding to the analysed mt-mRNA or T7 *in vitro* transcribed RNA probes corresponding to appropriate mt-tRNAs in 7% SDS and 0.25 M sodium phosphate buffer (pH 7.6), and washed with SSC (containing 0.1% SDS) five times for 20 minutes. The membrane was exposed to a storage phosphor screen (GE Healthcare), visualized using a Typhoon phosphorimaging system and quantified using ImageJ (http://imagej.nih.gov/ij).

Measurements of RNA steady-state levels by reverse transcription quantitative PCR (RT-qPCR) was performed as described previously (8). Briefly, RNA was extracted with TRIzol reagent (Thermo Fisher Scientific) and treated with TURBO DNase (Ambion) according to manufacturer's instructions. 1 μg RNA was reverse transcribed using Omniscript RT kit (Qiagen) with 0.5 μM random hexamers and 0.5 μM oligo dT primers. RT-qPCR primer and probe sequences can be found in Supplementary Table S1.

## RESULTS

### YbeY is a mitochondrial protein with a conserved endonuclease domain

**Figure 1.**
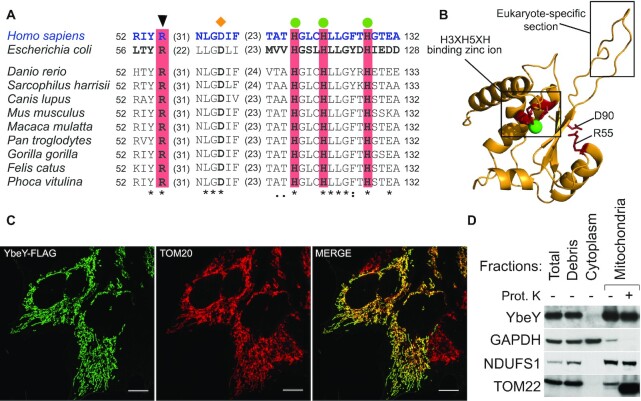
Sequence/structure conservation and mitochondrial localization of human YbeY. (**A**) Alignment of protein sequences of the orthologues of YbeY. The catalytically-important RNA-binding arginine is indicated a black triangle above. Green circles indicate histidine residues from the zinc ion-binding H3H5XH motif. An orange diamond above indicates the D90 required for the interaction with ribosomal SSU component S11. Colons and dots denote chemical similarity between the sequences; asterisks indicate identical residues. (**B**) Homology modelling of the human YbeY using *E. coli* YbeY as a template (PDB: 1XM5) superimposed on each other: human YbeY (Yellow), conserved catalytically important residues (red) and zinc ion (green). Eukaryote-specific insertion (Gly75-Pro83 in *H. sapiens*) is indicated. (**C**) FLAG-tagged YbeY was transiently expressed in HeLa cells and visualized using anti-FLAG antibody (green) and the mitochondrial outer membrane translocase, TOM20 (red). Co-localization is shown in yellow. The white scale bar at the bottom right depicts 10 μm. (**D**) Subcellular fractionation of HEK293T cells shows YbeY protein in the same fraction as NDUFS1 (mitochondrial inner membrane) and TOM22 (mitochondrial outer membrane, truncated by proteinase K), but not with GADPH (cytoplasm)

Human YbeY is an uncharacterized protein that is encoded by the *C21orf57* gene. It belongs to the UPF0054 protein family (PFAM accession number: PF02130) which contains a highly conserved zinc ion-coordinating consensus motif H3XH5XH (56). The bacterial homolog is an active endoribonuclease and contains the RNA-binding Arg59 residue and the histidine triad (34,40). Sequence alignment from multiple species (Figure 1 and Supplementary Figure S1) and homology modelling of human YbeY, using the *Escherichia coli* protein as the template, revealed strong conservation, especially of the aforementioned active site residues, suggesting that human YbeY is an active RNase (Figure 1B). In addition, the previous analysis showed that *E. coli* Asp85 (Asp90 *Homo sapiens*), found in the beta-sheet outside the active site and required for the interaction of YbeY with the ribosomal small subunit component, S11, is also conserved. Mutation of these residues has been shown to disrupt nuclease activity of the protein and rRNA maturation (42) (Supplementary Figure S1). Further comparison of the two homologs also shows that human YbeY has a nine amino acid insertion (Gly75–Pro83) between two conserved beta sheets which is conserved in other eukaryotic species (Supplementary Figure S1) and may be responsible for additional protein interactions and additional functions in the mitochondria (Figure 1B) (42).

YbeY has been previously predicted to be mitochondrially localized by large-scale databases, MitoCarta and MitoMiner 4.0 (57,58). Furthermore, large-scale CRISPR/Cas9 screen has revealed that YbeY is vital for OXPHOS function (59). In accordance with these results, *in silico* analysis of the subcellular localization of the protein by multiple online tools predicted a high probability of mitochondrial localization: Predotar (60)—0.87, TargetP (61)—0.86, and Mitoprot (62)—0.96. To confirm these predictions, we used immunostaining that showed a co-localization of transiently-expressed FLAG/Strep2-tagged YbeY to the mitochondria in HeLa cells (Figure 1C). We also used subcellular fractionation and immunoblotting to localize endogenous YbeY in the cell. YbeY co-fractionated with inner membrane-localised Complex I subunit, NDUFS1 (Figure 1D). To assess, if YbeY co-localizes within mitochondrial RNA granules (MRGs), where processing of nascent RNA occurs, immunocytochemistry was performed using antibodies against a MRG marker GRSF1 (63). While the MRGs appeared as distinct puncta, YbeY-FLAG/Strep2 was distributed throughout the mitochondria (Supplementary Figure S2). However, the diffuse spread of YbeY could be attributed to over-expression of the protein or due to multiple functions of the protein in the mitochondria. Taken together, this confirms that YbeY is a mitochondrial protein and may not co-localize within MRGs.

### YbeY is required for mitochondrial translation

**Figure 2.**
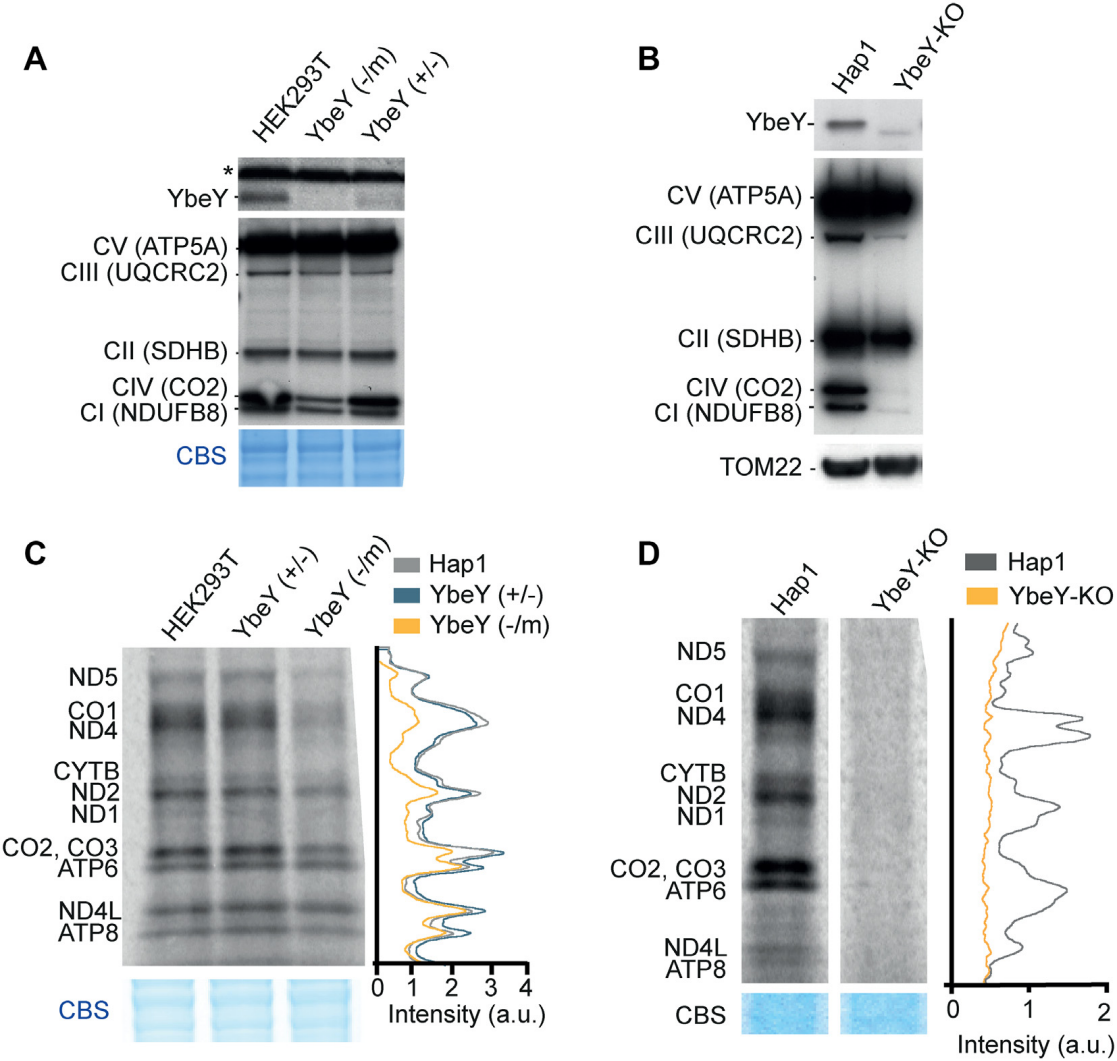
Reduced mitochondrial gene expression and growth in cells lacking YbeY. (**A**) Analysis of steady state levels of endogenous YbeY and OXPHOS subunits in HEK293T parental, with cells that have 1 functional copy of *YBEY* gene (+/–) and YbeY-deficient (-/m) cells. The asterisk denotes an unrelated cross-reacting protein band. Coomassie Blue staining (CBS) was used as loading control. (**B**) Analysis of steady state levels of endogenous YbeY and OXPHOS subunits in Hap1 parental and YbeY-KO cells. TOM22 was used as a loading control. (**C**) Metabolic labelling of mitochondrial translation products with [^35^S]-methionine in HEK293T parental cells, YbeY (+/–) and YbeY (–/m) cell lines. Coomassie Blue staining (CBS) was used as loading control. (**D**) *De novo* protein synthesis of Hap1 parental cells and YbeY-KO cells. Coomassie Blue staining (CBS) was used as loading control.

In order to investigate the role of YbeY, we inactivated its expression in human cells. YbeY-deficient HEK293T cell lines were generated using zinc finger nuclease (ZFN) targeting exon 2, whereas YbeY knockout Hap1 cells were created by CRISPR/Cas9. We found that HEK293T shows a high degree of polyploidy at the *YbeY* locus of chromosome 21. One of the investigated HEK293T cell lines with modified *YbeY* locus contained two out-of-frame deletions, one start-loss deletion and one allele with a 12 bp deletion and a change in 4 amino acids. This cell line is hereby referred to as YbeY (–/m) (Supplementary Figure S3A). The second HEK293T cell line with YbeY locus edited by the ZFN contained the three alleles with indels which led to premature stop codons, one allele contained an in-frame deletion leading to a loss of two amino acids and one allele had unmodified sequence. We named this HEK293T mutant YbeY (+/–) (Supplementary Figure S3B). We also used a near haploid, Hap1, YbeY knockout (YbeY-KO) cell line that contained a 31 bp insertion and 1bp mutation (Supplementary Figure S3C) which produces a premature stop codon and leads to an 80 amino acid truncated protein. All three YbeY-deficient cell lines exhibited severely depleted levels of endogenous protein. In all three cell lines, the expected truncated proteins were not observed in the western blots possibly due to nonsense-mediated mRNA decay or loss of the relevant epitope for antibody recognition. A non-specific cross-reacting protein band is observed in the HEK293T cell lines that is not present in the Hap1 cell line western blot (Figure 2A, B). This is attributed to the difference in gene expression profiles in the different cell lines. A lower molecular weight band is observed in YbeY-KO western blot against native YbeY protein. This is a non-specific cross-reacting protein that is not the correct size to be the truncated protein (Figure 2B). We believe that this is a YbeY knockout-specific product of altered gene expression.

Next, we intended to study the OXPHOS function and mitochondrial translation upon ablation of YbeY. Western blot analysis showed a strong reduction in the steady state levels of Complex I and IV subunits in YbeY(–/m) HEK293T cells (Figure 2A). A much stronger effect was observed in the complete knockout cell line where, in addition to Complex I and IV, a Complex III subunit was also depleted, suggesting a defect in synthesis of the OXPHOS subunits in the absence of YbeY. Consistent with this result, cell growth was compromised when YbeY-deficient HEK293T cells were grown in media containing galactose as the sole carbon source, forcing them to rely on mitochondrial ATP production (Supplementary Figure S4A). The growth of YbeY-KO Hap1 cells was compromised on standard, glucose-containing medium while the cells failed to grow on medium containing galactose as the sole carbon source (Supplementary Figure S4B). The effect of YbeY depletion on mitochondrial translation was assessed using *in vivo* incorporation of [^35^S]-methionine into newly synthesiszed proteins. A global mitochondrial translation defect was observed in the YbeY-KO cells, while a reduction of protein synthesis was observed in the YbeY(–/m) cells (Figure 2C, D). Moreover, in YbeY-deficient HEK293T cells, the higher molecular weight translation products were affected to a greater degree than the lower molecular weight products (Supplementary Figure S4C). This preferential depletion of longer translation products may be a result of a detrimental effect on translation elongation. This effect of defective RNA processing on mitochondrial translation has been previously observed and suggests that it is not specific to the lack of YbeY but rather a result of translation dysfunction (51). To confirm that the strong mitochondrial translation defect observed in the YbeY-KO cells was not a consequence of decreased cytoplasmic translation, we repeated the labelling experiment without any translation inhibitors or in the presence of chloramphenicol (CAP) that inhibits mitochondrial translation. We observed that global translation was not affected by the ablation of YbeY (Supplementary Figure S4D, lanes 1 and 2). The observed decrease of cytoplasmic translation in YbeY-KO was attributed to the prolonged exposure to the competitive inhibitor CAP required to completely inhibit mitochondrial translation (Supplementary Figure S4D, lanes 3–4). This showed that the loss of translation was mitochondria-specific and not caused as a by-product of reduced cytoplasmic translation. Taken together, these result show that YbeY is indispensable for mitochondrial translation in human cells.

### YbeY is required for accurate mt-tRNA^Ser(AGY)^ processing

**Figure 3.**
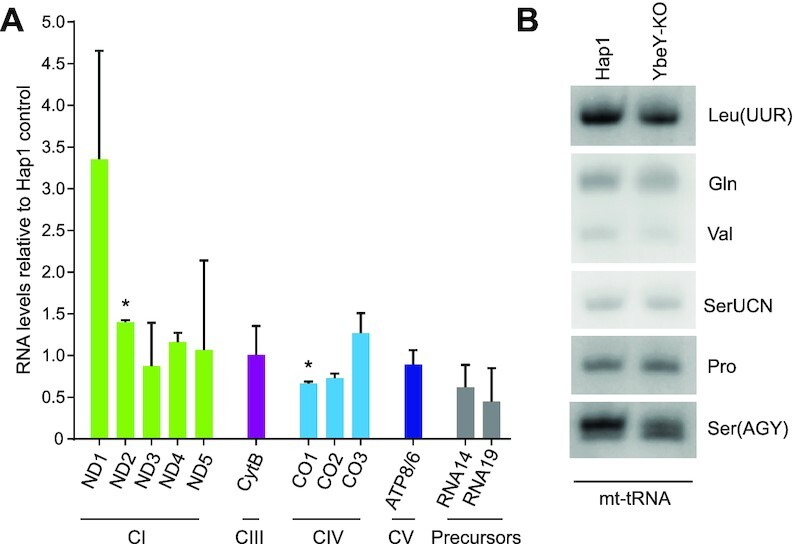
Steady state levels of mitochondrial RNA in YbeY depleted cells. (**A**) Steady-state levels of mitochondrial mRNAs quantified by northern blotting in Hap1 control and YbeY knockout Hap1 cells grown in standard IMDM medium. 5S RNA was used as a loading control. Quantification of steady state levels was performed using Image J and normalized to the 5S RNA loading control and values from Hap1 control cells. *P < 0.05, One sample t-test, Error bars = 1 SD. (**B**) High resolution northern blot analysis of mitochondrial tRNAs from Hap1 control and YbeY knockout Hap1 cells. *n* = 2.

Given the strongest effect on mitochondrial translation and a complete ablation of YbeY in the Hap1 YbeY-KO cells, this cell line has been taken forward for further investigations. Next, we set out to investigate if human YbeY has a role in controlling mitochondrial transcript steady-state levels in YbeY-KO Hap1 cells. Northern blot analysis showed that there was no substantial difference in the levels of mitochondrial mRNAs relative to Hap1 control cells (Figure 3A, Supplementary Figure S5). Any observed increase in mt-mRNAs coding for Complex I subunit ND1 and ND2 steady state levels and a decrease in mt-mRNAs encoding Complex IV subunit CO1 and CO_2_ (Figure 3A, Supplementary Figure S5) was not attributed to YbeY deficiency, as a similar compensatory phenotype has been often observed in cells responding to defective mitochondrial translation (51,64–66).

**Figure 4.**
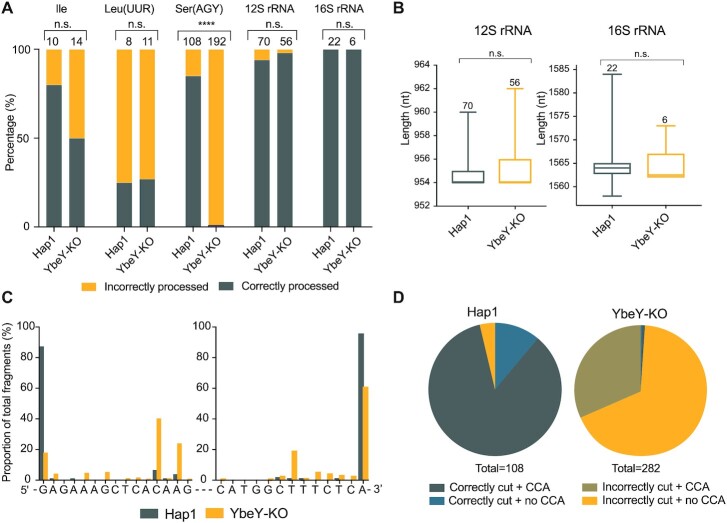
YbeY depletion causes defects in mt-tRNA^Ser(AGY)^ end processing. (**A**) Results of circularization RT-PCR analysis of mt-tRNA^Ile^, mt-tRNA^Leu(UUR)^, mt-tRNA^Ser(AGY)^, 12S mt-rRNA and 16S mt-rRNA from Hap1 control and YbeY knockout Hap1 cells. DNA bands obtained in the circularization RT-PCR reaction were analysed by Sanger sequencing upon cloning. Number of clones analysed in each sample is given above each bar. Analysis of significance was calculated using Fisher's exact test. ^****^P < 0.0001. (**B**) The length of the 12S and 16S mt-rRNAs calculated from the 5′ and 3′ ends. The lines indicate the minimum and maximum values and the edges of the boxes indicate the upper and lower quartile. The middle line indicates the median. The number of data points are written above the box and whisker plots. Significance was assessed using an unpaired Student's *t*-test (n.s., not significant). (**C**) Mapping of the sequence of the 5′ and 3′ ends of circularized mt-tRNA^Ser(AGY)^ in Hap1 parental and YbeY knockout Hap1 cells. The 5′ and 3′ ends were plotted independently as a percentage of the total 5′ and 3′ reads sequence, respectively. (**D**) Proportion of mt-tRNA^Ser(AGY)^ that are correctly or incorrectly cleaved from the primary transcript at the expected ends and modified (or not) to have a 3′ CCA.

Further analysis of mitochondrial tRNA in YbeY-KO Hap1 cells using high resolution northern blotting showed a potential steady-state decrease and/or processing defect of mt-tRNA^Ser(AGY)^ relative to other mt-tRNAs analysed (Figure 3B). Given that bacterial YbeY is predominantly involved in rRNA end processing, we wanted to investigate the effects of YbeY knockout on the 5′ and 3′ ends of the mt-rRNAs and mt-tRNAs to see if YbeY plays a similar role in the mammalian mitochondria. To do so, we used circularization RT-PCR to ligate the 5′ end of the RNA molecule to the 3′ end and sequence the junction. Of the RNAs analysed, no significant difference was observed in the 5′ and 3′ processing of mt-tRNA^Ile^, mt-tRNA^Leu(UUR)^, 12S mt-rRNA and 16S mt-rRNA when Hap1 parental cells and YbeY knockout Hap1 cells were compared (Figure 4A, B). However, in YbeY knockout Hap1 cells, only ∼1% of the mt-tRNA^Ser(AGY)^ molecules were correctly cleaved from the primary transcript and modified to add the 3′ CCA, as compared to the ∼85% in the control Hap1 cells (Figure 4A). Sequence analysis of the mt-tRNA processing in the YbeY knockout Hap1 cells revealed that only ∼20% of mt-tRNA^Ser(AGY)^ were correctly processed from the 5′ terminus and ∼64% from the 3′ end (Figure 4C, D). This confirms that YbeY is required for the correct end processing of mt-tRNA^Ser(AGY)^. However, due to the limited number of RNAs that could be studied and the limitations of the circularization RT-PCR method, it is possible that YbeY is also involved in the processing of other mitochondrial tRNAs.

### YbeY interacts with mitochondrial ribosomal components

**Figure 5.**
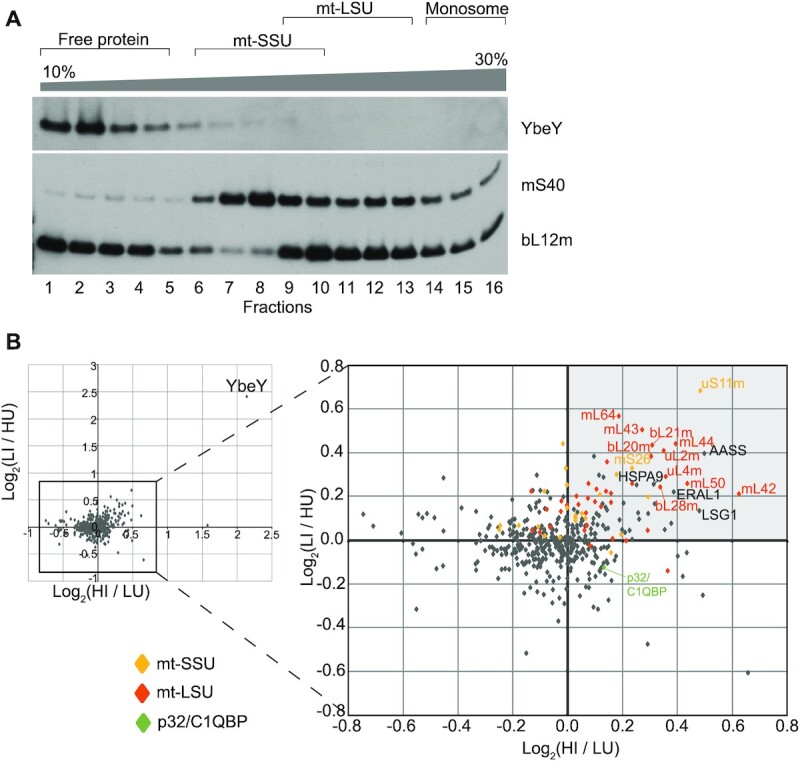
Human YbeY interacts with uS11m. (**A**) Analysis of the interaction of endogenous YbeY with the mitochondrial ribosome using isokinetic sucrose gradient fractionation with mitochondria from Hap1 parental cells, and western blotting. Fractions containing unassembled free protein, the subunits at various stages of assembly, and the associated mitoribosome are indicated above. Antibodies against mS40 and bL12m were used to identify the fractions containing the small and large mitoribosome subunits, respectively. (**B**) Immunoaffinity purification of FLAG-tagged YbeY was performed from doxycycline induced or uninduced, differentially labelled cells (please see Materials and Methods). Base two logarithm (Log_2_) of the ratio of peptide abundance in induced cells (I) to peptide abundance in uninduced (U) cells were plotted on the X and Y axes from reciprocal experiments that used heavy (H) or light (L) amino acid labelled cells. Log_2_(HI/LU) indicates the base two logarithm of the ratio of peptide abundance from doxycycline induced cells labelled with ^15^N and ^13^C L-arginine and L-lysine to peptide abundance of uninduced cell labelled with ^14^N and ^12^C l-arginine and l-lysine. Log_2_(LI/HU) indicates the base two logarithm of the ratio of peptide abundance from doxycycline induced cells labelled with ^14^N and ^12^C l-arginine and L-lysine to peptide abundance of uninduced cell labelled with ^15^N and ^13^C l-arginine and l-lysine. Proteins in the top right quadrant (indicated in light grey) are those that were enriched in the YbeY-overexpressing HEK293T cell line in both labelling experiments and proteins in the lower left quadrant are those that are more abundant in the control cell line. Mt-SSU components (yellow diamonds), mt-LSU components (orange diamonds), p32/C1QBP (green diamond) and other proteins (grey diamonds).

In order to study a potential interaction of human YbeY with the mitoribosomal subunits, we used western blot analysis after sucrose gradient fractionation of human HEK293T cells. A majority of the endogenous YbeY was found in the ‘free protein’ fractions (Figure 5A, fractions 1–4). However, a small proportion co-migrated with the mt-SSU, suggesting potential weak interaction of YbeY with this subunit (Figure 5A, fractions 6–9). Next, we performed YbeY pull-down experiments, upon overexpression of a FLAG/Strep2-tagged version of the protein (Supplementary Figure S6A), followed by identification of potential interactors by mass spectrometry upon resolving the eluates on SDS-PAGE (Supplementary Figure S6B). We only detected the mitochondrial chaperones, p32/C1QBP and HSPA9/GRP75, being pulled-down in this experiment (Supplementary Figure S6B), which were observed previously as common contaminants in this type of experiment (19,49). To study the potential YbeY-p32/C1QBP interaction further, we performed a reciprocal experiment with FLAG/Strep2-tagged p32/C1QBP. We did not detect YbeY being pulled-down with p32/C1QBP used as a bait (Supplementary Figure S6C). Next, in order to identify interactors of human YbeY with a higher sensitivity, we used quantitative SILAC-based immunoaffinity purification of FLAG-tagged YbeY in a doxycycline-inducible HEK293T cell line, using uninduced cells as control. Since we did not see a reciprocal non-SILAC pull-down, we decided to remove the Strep2 tag in case it was somehow contributing to the observed YbeY-p32/C1QBP interaction. The dox-induced expression levels of YbeY-FLAG/Strep2 and YbeY-FLAG were comparable (Supplementary Figure S6A). Reciprocal SILAC experiments identified various mt-SSU and mt-LSU components being pulled-down with YbeY. The strongest interactor of YbeY appeared to be uS11m. Other weaker interactors of YbeY included ERAL1 and several mitoribosomal proteins such as uL2m, uL4m, bL20m, bL21m, mL42, mL43, mL50, mL64 and mS26 (Figure 5B, Supplementary Figure S6D, E, Supplementary Dataset 1). The interaction with uS11 and ERAL1 is highly conserved in bacteria and vital for the maturation of the small subunit (67). Of note, while HSPA9/GRP75 was found to be slightly enriched in the YbeY-FLAG SILAC-based, quantitative immunoaffinity purification experiments, there was no substantial enrichment of p32/C1QBP (Figure 5B). On the whole, these results show conserved interactions of YbeY with the ribosome and, hence, a potentially conserved role in ribosome biogenesis between bacteria and human mitochondria.

### Loss of YbeY impairs mitoribosome assembly

**Figure 6.**
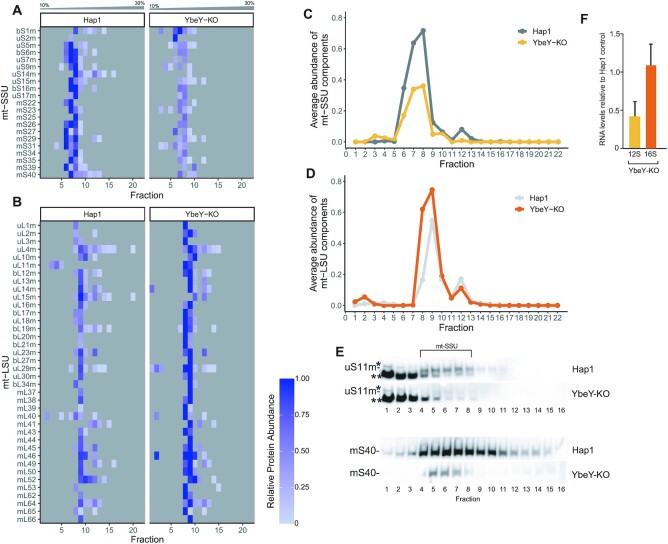
Assembly of mt-SSU is perturbed in YbeY knockout Hap1 cells. (**A, B**) Quantitative mass spectrometry analysis of mt-SSU (A) and mt-LSU (B) in the parental and YbeY KO Hap1 cells. The samples were subjected to quantitative density gradient analysis by mass spectrometry (qDGMS) and raw data were processed with Proteome Discoverer and further analysed in the ComPrAn R package (52). Representative peptides for each protein are selected on the basis of being present in the highest number of fractions, for both labelling states considered together. Thus, for any given protein, the representative peptide is the same for both labelling states. A representative peptide was not selected for proteins that do not have shared peptides or are not present in both labelling states—these proteins are not shown. Each row represents a different mitoribosomal component. Each column represents the fractions taken from the sucrose gradient going from 10% sucrose in fraction 1 to 30% sucrose in fraction 22. Grey squares represent the absence of detection of a protein in a given fraction. (**C, D**) Quantitative comparison of the average abundance of the mt-SSU (C) and mt-LSU (D) components in the sucrose gradient in YbeY-KO and Hap1 WT cells. (**E**) Analysis of the abundance of uS11m and mS40 in the mt-SSU fractions using isokinetic sucrose gradient fractionation and western blotting in YbeY-KO and Hap1 WT cells. * and ** denote unspecific protein bands detected by anti-uS11m antibody. (**F**) RT-qPCR determination of transcripts levels in Hap1 YbeY-KO cells grown in IMDM medium for SILAC. Steady-state levels of 12S and 16S mt-rRNA were normalized to GAPDH and are presented as values relative to Hap1 wild-type control cells. *n* = 3. Error bars indicate SEM. *P* = 0.066—statistical analysis was carried out using a two-tailed Student's t-test.

In *E. coli*, YbeY is responsible for ribosomal quality control. With the assistance of exonuclease RNase R, bacterial YbeY degrades rRNA from late stage ribosome assembly intermediates with defective small subunits (34,40). Hence, we intended to investigate the effect of YbeY depletion on the mitoribosome integrity. To quantitatively assess changes in composition and integrity of the mitoribosome in the YbeY knockout Hap1 cells, we used our recently developed complexome profiling pipeline that involves quantitative density gradient analysis by mass spectrometry (qDGMS), in combination with the data analysis R package – ComPrAn (52). The qDGMS experiments revealed that levels of assembled mt-SSU were decreased by ∼50% in the YbeY knockout cells (Figure 6A, C, Supplementary Dataset 2), while mt-LSU was generally more abundant in the knockout cells relative to the control Hap1 cells (Figure 6B, D, Supplementary Dataset 2). Next, we focused on the analysis of the main YbeY interactor uS11m. We did not detect any uS11m peptides in the YbeY-KO samples in the qDGMS experiment, however uS11m was detected in the control (Supplementary Figure S7, Supplementary Dataset 3). This result is consistent with a very low level of uS11m (below MS detection limit) in the assembled mt-SSU upon ablation of YbeY. To verify this, we performed western blot analysis of YbeY-KO Hap1 cells and indeed observed a severe decrease in the steady-state levels of uS11m (Supplementary Figure S8A). This analysis also showed a decrease in the steady-state levels of other mt-SSU components, while the steady-state levels of mt-LSU remained largely unchanged, as compared to a control (Supplementary Figure S8A). To further verify the effect of YbeY inactivation on the assembly of uS11m into mt-SSU, we performed western blot analysis upon sucrose gradient fractionation using Hap1 parental and YbeY-KO Hap1 cells. We observed a severe decrease in abundance of uS11m in the fraction corresponding to mt-SSU (Figure 6E). Consistent, with the qDGMS results, this experiment also showed the general reduction in the abundance of the assembled mt-SSU (Supplementary Figure S8B, C). Additional analysis also revealed a substantial decrease in 12S mt-rRNA steady-levels when the YbeY-KO Hap1 cells were grown in SILAC IMDM medium, consistent with the qDGMS data (Figure 6F). Of note, this decrease in steady-state levels of 12S mt-rRNA was not observed for the YbeY-KO Hap1 cells cultured in standard IMDM medium (Supplementary Figure S9). Taken together these experiments indicate that the loss of YbeY reduces the assembly or stability of mature mt-SSU leading to mitochondrial translation impairment.

## DISCUSSION

The translation of the mitochondrially-encoded polypeptides requires the coordinated action of numerous factors. Many of these factors act at the RNA level such as cleaving the primary transcript, chemically modifying mt-tRNAs and mt-rRNAs, or chaperoning the mt-rRNA into the correct conformation (68). Here, we have identified YbeY, a mitochondrially-localized protein with a conserved endonucleolytic domain. Deletion of the gene led to decreased steady state levels of the OxPhos complexes which have mitochondrially-encoded components. Moreover, analysis of *de novo* mitochondrial protein synthesis showed that YbeY depletion had a detrimental effect on ribosome activity.

The main catalytic residues involved in endonucleolytic cleavage, are conserved in mammalian YbeY suggesting the conservation of its endonucleolytic function (42). Moreover, YbeY has been shown to be an active RNase in various bacterial species (34,36,47) and in plant chloroplasts (48).In *E. coli*, similar to the mitochondria, the ribosomal RNAs are initially transcribed within a primary transcript and are later released through multiple hierarchical cleavage events. Bacterial YbeY is responsible for the 5′ and 3′ end maturation of the 16S (small subunit rRNA) precursor, the 5′ end maturation of the 23S (large subunit rRNA) precursor and the final steps of 5S rRNA maturation (40). In the mammalian mitochondria, however, the excision of the flanking mt-tRNAs by RNase P and ELAC2 produces the mt-rRNAs ready for mitochondrial assembly. Thus, to investigate if the translation defect was due to errors in RNA processing, we investigated the effect of YbeY depletion on RNA steady state levels. We observed no substantial differences in mRNA steady-state levels or processing, however, there was a noticeable impact on mt-tRNA^Ser(AGY)^, and mt-tRNA^Leu(UUR)^ to a lesser extent. Analysis of the 5′ and 3′ termini of the mt-tRNAs showed a severe deficiency in accurate post-transcriptional processing of mt-tRNA^Ser(AGY)^ from the primary transcript in YbeY-deficient cells. However, there are various drawbacks to the circularization RT-PCR that we used to analyse the 5′ and 3′ termini of the mt-tRNAs which may have led to a large variation in data points acquired for the different tRNAs. Any misprocessed mt-tRNA smaller than the size of the primers was not likely to be detected. Moreover, the ligation reaction would be biased towards misprocessed RNAs with weaker secondary structures depending on their size and composition. Therefore, the proportion of misprocessed to correctly processed RNAs would be higher than that present in living cells, which may have influenced the proportion of misprocessed mt-tRNAs observed for mt-tRNA^Ile^ and mt-tRNA^Leu(UUR)^. Normally, the 5′ end of RNAs in the mitochondrial primary transcripts is processed by RNase P before the 3′ end processing by RNase Z (3,29). However, previous work has suggested that mt-tRNA^Ser(AGY)^ is not processed completely at the 5′ end by RNase P (69). The authors suggested that the release of the 5′ end of the mt-tRNA is a consequence of the 3′ endonucleolytic processing of the upstream mt-tRNA^His^, while the presence of the downstream mt-tRNA^Leu(UUR)^ was dispensable for mt-tRNA^Ser(AGY)^ 3′ end processing (69). Furthermore, transcriptomic analysis of RNase P-deficient MRPP3 knockout mice show that mt-tRNA^Ser(AGY)^ is not affected by the lack of RNase P (29) while the presence of ELAC2 is required for the processing of mt-tRNA^Ser(AGY)^ as loss of the enzyme leads to accumulation of unprocessed precursors (70). Here, we demonstrate that in the absence of YbeY, steady state levels of mt-tRNA^Ser(AGY)^ are reduced, and the 5′ and to a lesser extend the 3′ ends of mt-tRNA^Ser(AGY)^ are erroneously processed. However, a small proportion of correctly processed mt-tRNAs are still produced and can be used in translation. We suggest that YbeY may work in conjunction with ELAC2 to accurately cleave the mt-tRNA at the 3′ end of the upstream mt-tRNA^His^ and at the 3′ end of mt-tRNA^Ser(AGY)^. However, we do not observe a direct interaction between YbeY and ELAC2. It is unclear if YbeY directs tRNA processing through direct cleavage or with the assistance of additional factors. It is also possible that the tRNA is correctly processed by RNase P and ELAC2 but then undergoes exonucleolytic cleavage in the absence of YbeY. Moreover, due to the limited number of mt-tRNAs studied, it cannot be excluded that YbeY facilitates the processing of other mt-tRNAs.

In addition to RNA processing, the bacterial YbeY degrades rRNA from late stage ribosomes with defective small subunits and as such is responsible for quality control (34,40). We show that, in the mammalian mitochondria, YbeY is crucial for the integrity of the small subunit. The knockout of YbeY leads to a depletion of mt-SSU as analysed by a quantitative proteomics approach and western blotting. The reduced steady-state levels of mt-SSU in the Hap1 cells lacking YbeY is accompanied with a concurrent decrease in steady-state levels of 12S mt-rRNA (Figure 6E), while the ends of 12S mt-rRNA are correctly processed (Figure 4, Supplementary Figure S8A). Of note, the reduction in 12S mt-rRNA was only seen in SILAC medium, containing dialysed foetal bovine serum, but not in standard medium. This intriguing growth condition-depended effect on RNA metabolism in mitochondria has been observed before, with culturing Hap1 cells in SILAC medium generally reducing mt-RNA steady-state levels (8). It would be interesting to compare mt-RNA half-life in these growth conditions. Also, caution should be exercised when studying mitochondrial gene expression in Hap1 cells cultured in these different media. When our manuscript was under review, Summer *et al.* (71) reported that YbeY is involved in the biogenesis of mt-SSU. Consistent with our result, Summer *et al.* showed that YbeY is a mitochondrial protein and its inactivation in HEK293T-REx cells leads to a decrease in steady-state levels of mt-SSU protein components, as shown by western blotting, and reduction of 12S mt-rRNA, examined by RT-qPCR and RNASeq. On the other hand, and in line with our data, the analysis of mt-LSU components and 16S mt-rRNA showed no reduction between control and HEK293T-REx YbeY-KO cells, supporting our conclusion on YbeY being involved in the biogenesis of mt-SSU.

Analysis of interactors of the human YbeY showed that it interacts with uS11m. A weaker interaction of YbeY has been observed for ERAL1. Both uS11m and ERAL1 are key for mt-SSU biogenesis, suggesting a role for YbeY in mitoribosomal biogenesis similar to its role in bacteria. The GTPase ERAL1 is a 12S mt-rRNA chaperone and mt-SSU assembly factor (21). In bacteria, the homolog of YbeY interacts with ribosomal protein S11 (homolog of mitoribosomal uS11m) through a conserved four stranded beta sheet, and GTPase Era (homolog of ERAL1). However, the interaction with Era is facilitated indirectly through its interaction with S11, which could explain the observed weak interaction of ERAL1 with YbeY. A disruption of this YbeY-S11 interaction in bacteria led to an accumulation of 16S small subunit rRNA precursors and a resultant increase in susceptibility to stress (67). The residues involved with the YbeY-S11 interaction are conserved in human YbeY (67). Therefore, this suggests that, similar to its bacterial homolog, YbeY associates with ERAL1 indirectly through uS11m. Both ERAL1 and its bacterial homolog Era binds stem-loop structures at the 3′ end of the SSU rRNA (21,22). In mammalian 12S mt-rRNA, this stem-loop region contains two adenines that are methylated by TFB1M, and this modification is necessary for ERAL1 activity (22). The progression of mt-SSU assembly requires the removal and targeted degradation of ERAL1 from this site (72). Structural analysis of the mitochondrial ribosome suggests that, similar to the bacterial ribosome, the uS11m component is assembled at the 3′ end of the SSU mt-rRNA, at the site of ERAL1 interaction.

Mitochondrial uS11m has extensive connections with the 12S mt-rRNA and binds independently to other protein clusters during the assembly process (31). However, previously published pulse-chase quantitative proteomic analysis of small subunit assembly in bacteria and human cells have demonstrated that while the mitochondrial uS11m subunit is an early binding protein, S11 is one of the last proteins to be assembled into the bacterial SSU (31,73). In addition to uS11m, YbeY interactors also included mS26, mL64, uL2 and early-assembly mt-LSU clusters, bL20m-bL21m-mL42-mL43-mL44 and uL4m-mL50 (31). Mapping these components onto the mitoribosomal structures show that these subcomplexes form a continuous belt of proteins around the large subunit and have extensive contacts with the 16S mt-rRNA (Supplementary Figure S6D, E). However, these interactions with the mt-LSU subcomplexes are weak and might not be direct, but rather secondary interactions via rRNA or other proteins. These data are consistent with the recent study by Summer *et al.* (71) who also report YbeY interaction with mt-LSU components in the FLIM-FRET (uL24m, bL20m, uL4) and YbeY pull-down (bL20m, bL21m, uL22m, uL23m, uL24m, bL28m, mL50) experiments. Many of these mt-LSU proteins are common for both studies (uL4, bL20m, bL21m, uL24m, bL28m, mL50). It would be interesting to follow up these observations, as they may point towards a link between mt-SSU and mt-LSU biogenesis.

The related study by Summer *et al.* (71) reported p32/C1QBP as a YbeY-interacting protein. P32/C1QBP is a highly abundant protein reported to localize in various cellular compartments, including the nucleus, nucleoli, cytosol and mitochondria (74–76). This moonlighting protein has been implicated in inflammation and infection processes, regulation of apoptosis, nuclear transcription, pre-mRNA splicing, autophagy and tumorigenesis, as well as to have an RNA chaperone role in mitochondria (77–81). Initially, we pulled-down p32/C1QBP upon overexpression of YbeY-FLAG/Strep2 (Supplementary Figure S6B). However, the FLAG tagged version of p32/C1QBP did not co-purify with YbeY in a reciprocal experiment (Supplementary Figure S6C). The latter result, however, could be related to a high abundance of p32/C1QBP and in the absence of high levels of YbeY overexpression there may not be enough endogenous YbeY protein to be readily detected by mass spectrometry. Alternatively, the FLAG tag may interfere with formation of the p32/C1QBP trimer or with its interaction with YbeY. Importantly, p32/C1QBP was not found to be enriched in the YbeY SILAC-based, quantitative immunoaffinity purification experiments (Figure 5B). It is possible that expression and/or steady-state level of p32/C1QBP is stimulated by mitochondrial stress upon overexpression of YbeY, consistent with recent findings on compensatory mechanisms in muscle fibers struggling with metabolic stress (82). Substantial upregulation of p32/C1QBP, in this case, would cause a non-specific enrichment in the pull-down experiment and would be subsequently identified by mass spectrometry. However, enrichment of p32/C1QBP is not observed in both (HI/LU and LI/HU) quantitative proteomic experiments. Indeed, in one of the experiments, p32/C1QBP levels are more abundant in the control cell line, hence p32/C1QBP is not identified as enriched. However, we also cannot exclude the possibility that the inconsistences in SILAC and non-SILAC YbeY pull-down results stem from the different tags used (non-SILAC IP: FLAG/Strep2-tag; SILAC IP: FLAG-tag). On the whole, the role of p32/C1QBP-YbeY interaction in mt-SSU assembly proposed by Summer *et al.*, based mainly on immunoaffinity purification and indirect FLIM-FRET analyses in the conditions of YbeY overexpression, warrants further investigations.

Fractionation experiments performed by others are consistent with early mt-LSU assembly and a majority of mt-SSU assembly occurring in the nucleoid (mtDNA containing fractions) after which mitoribosome assembly continues in MRGs, the latter being based on co-localization experiments (19,28,30–32). Our data suggests that YbeY contributes to late assembly of mt-SSU. However, previously published proteomic analysis of nucleoid-associated proteins did not identify YbeY as a constituent of nucleoids (28) and our results indicate that YbeY may not localize to MRGs. The diffuse distribution of YbeY in the mitochondrial matrix may be as a result of overexpression of the protein or its multiple functions in mitochondria. Many mitochondrial RNA binding proteins, such as MRPP1, MRPP2 and NSUN4, have multiple independent functions in RNA modification (17,83–85). Our results have demonstrated that YbeY, similarly performs multiple independent functions in the mammalian mitochondria. On the other hand, these results may point towards a possible functional link between the post-transcriptional processing of the primary transcript, mitoribosomal biogenesis and translation suggested before (29). Moreover, our results also highlight a possible connection between the assembly pathways of the large and small mitoribosomal subunits.

## Supplementary Material

gkab404_Supplemental_Files

## Notes

Present address: Joanna Rorbach, Department of Medical Biochemistry and Biophysics, Karolinska Institutet, Biomedicum, Solnavägen 9, 171 65 Solna, Sweden.

## References

[1] Anderson S. , Bankier A.T., Barrell B.G., de Bruijn M.H., Coulson A.R., Drouin J., Eperon I.C., Nierlich D.P., Roe B.A., Sanger F. et al. Sequence and organization of the human mitochondrial genome. Nature. 1981; 290:457–465. 7219534 10.1038/290457a0

[2] Ojala D. , Montoya J., Attardi G. tRNA punctuation model of RNA processing in human mitochondria. Nature. 1981; 290:470–474. 7219536 10.1038/290470a0

[3] Brzezniak L.K. , Bijata M., Szczesny R.J., Stepien P.P. Involvement of human ELAC2 gene product in 3′ end processing of mitochondrial tRNAs. RNA Biol. 2011; 8:616–626. 21593607 10.4161/rna.8.4.15393

[4] Holzmann J. , Frank P., Loffler E., Bennett K.L., Gerner C., Rossmanith W. RNase P without RNA: identification and functional reconstitution of the human mitochondrial tRNA processing enzyme. Cell. 2008; 135:462–474. 18984158 10.1016/j.cell.2008.09.013

[5] Amunts A. , Brown A., Toots J., Scheres S.H., Ramakrishnan V. Ribosome. The structure of the human mitochondrial ribosome. Science. 2015; 348:95–98. 25838379 10.1126/science.aaa1193 PMC4501431

[6] Greber B.J. , Bieri P., Leibundgut M., Leitner A., Aebersold R., Boehringer D., Ban N. Ribosome. The complete structure of the 55S mammalian mitochondrial ribosome. Science. 2015; 348:303–308. 25837512 10.1126/science.aaa3872

[7] Powell C.A. , Minczuk M. TRMT2B is responsible for both tRNA and rRNA m(5)U-methylation in human mitochondria. RNA Biol. 2020; 17:451–462. 31948311 10.1080/15476286.2020.1712544 PMC7237155

[8] Van Haute L. , Hendrick A.G., D'Souza A.R., Powell C.A., Rebelo-Guiomar P., Harbour M.E., Ding S., Fearnley I.M., Andrews B., Minczuk M METTL15 introduces N4-methylcytidine into human mitochondrial 12S rRNA and is required for mitoribosome biogenesis. Nucleic Acids Res. 2019; 47:10267–10281. 31665743 10.1093/nar/gkz735 PMC6821322

[9] Zaganelli S. , Rebelo-Guiomar P., Maundrell K., Rozanska A., Pierredon S., Powell C.A., Jourdain A.A., Hulo N., Lightowlers R.N., Chrzanowska-Lightowlers Z.M. et al. The pseudouridine synthase RPUSD4 Is an essential component of mitochondrial RNA granules. J. Biol. Chem. 2017; 292:4519–4532. 28082677 10.1074/jbc.M116.771105 PMC5377769

[10] Kotani T. , Akabane S., Takeyasu K., Ueda T., Takeuchi N. Human G-proteins, ObgH1 and Mtg1, associate with the large mitochondrial ribosome subunit and are involved in translation and assembly of respiratory complexes. Nucleic Acids Res. 2013; 41:3713–3722. 23396448 10.1093/nar/gkt079 PMC3616715

[11] Maiti P. , Antonicka H., Gingras A.C., Shoubridge E.A., Barrientos A. Human GTPBP5 (MTG2) fuels mitoribosome large subunit maturation by facilitating 16S rRNA methylation. Nucleic Acids Res. 2020; 48:7924–7943. 32652011 10.1093/nar/gkaa592 PMC7430652

[12] Lavdovskaia E. , Denks K., Nadler F., Steube E., Linden A., Urlaub H., Rodnina M.V., Richter-Dennerlein R. Dual function of GTPBP6 in biogenesis and recycling of human mitochondrial ribosomes. Nucleic Acids Res. 2020; 48:12929–12942. 33264405 10.1093/nar/gkaa1132 PMC7736812

[13] Kim H.J. , Barrientos A. MTG1 couples mitoribosome large subunit assembly with intersubunit bridge formation. Nucleic Acids Res. 2018; 46:8435–8453. 30085276 10.1093/nar/gky672 PMC6144824

[14] Lavdovskaia E. , Kolander E., Steube E., Mai M.M., Urlaub H., Richter-Dennerlein R. The human Obg protein GTPBP10 is involved in mitoribosomal biogenesis. Nucleic Acids Res. 2018; 46:8471–8482. 30085210 10.1093/nar/gky701 PMC6144781

[15] Maiti P. , Kim H.J., Tu Y.T., Barrientos A. Human GTPBP10 is required for mitoribosome maturation. Nucleic Acids Res. 2018; 48:7924–7943. 10.1093/nar/gky938 PMC6265488 30321378

[16] Rorbach J. , Boesch P., Gammage P.A., Nicholls T.J., Pearce S.F., Patel D., Hauser A., Perocchi F., Minczuk M. MRM2 and MRM3 are involved in biogenesis of the large subunit of the mitochondrial ribosome. Mol. Biol. Cell. 2014; 25:2542–2555. 25009282 10.1091/mbc.E14-01-0014 PMC4148245

[17] Camara Y. , Asin-Cayuela J., Park C.B., Metodiev M.D., Shi Y., Ruzzenente B., Kukat C., Habermann B., Wibom R., Hultenby K. et al. MTERF4 regulates translation by targeting the methyltransferase NSUN4 to the mammalian mitochondrial ribosome. Cell Metab. 2011; 13:527–539. 21531335 10.1016/j.cmet.2011.04.002

[18] Al-Furoukh N. , Goffart S., Szibor M., Wanrooij S., Braun T. Binding to G-quadruplex RNA activates the mitochondrial GTPase NOA1. Biochim. Biophys. Acta. 2013; 1833:2933–2942. 23933583 10.1016/j.bbamcr.2013.07.022

[19] He J. , Cooper H.M., Reyes A., Di Re M., Kazak L., Wood S.R., Mao C.C., Fearnley I.M., Walker J.E., Holt I.J. Human C4orf14 interacts with the mitochondrial nucleoid and is involved in the biogenesis of the small mitochondrial ribosomal subunit. Nucleic Acids Res. 2012; 40:6097–6108. 22447445 10.1093/nar/gks257 PMC3401442

[20] Kolanczyk M. , Pech M., Zemojtel T., Yamamoto H., Mikula I., Calvaruso M.A., van den Brand M., Richter R., Fischer B., Ritz A. et al. NOA1 is an essential GTPase required for mitochondrial protein synthesis. Mol. Biol. Cell. 2011; 22:1–11. 21118999 10.1091/mbc.E10-07-0643 PMC3016967

[21] Dennerlein S. , Rozanska A., Wydro M., Chrzanowska-Lightowlers Z.M., Lightowlers R.N. Human ERAL1 is a mitochondrial RNA chaperone involved in the assembly of the 28S small mitochondrial ribosomal subunit. Biochem. J. 2010; 430:551–558. 20604745 10.1042/BJ20100757 PMC2995420

[22] Uchiumi T. , Ohgaki K., Yagi M., Aoki Y., Sakai A., Matsumoto S., Kang D ERAL1 is associated with mitochondrial ribosome and elimination of ERAL1 leads to mitochondrial dysfunction and growth retardation. Nucleic Acids Res. 2010; 38:5554–5568. 20430825 10.1093/nar/gkq305 PMC2938226

[23] Tu Y.T. , Barrientos A. The human mitochondrial DEAD-Box protein DDX28 resides in RNA granules and functions in mitoribosome assembly. Cell Rep. 2015; 10:920–932. 25683708 10.1016/j.celrep.2015.01.033 PMC4534351

[24] Antonicka H. , Shoubridge E.A. Mitochondrial RNA granules are centers for posttranscriptional RNA processing and ribosome biogenesis. Cell Rep. 2015; 10:920–932. 25683715 10.1016/j.celrep.2015.01.030

[25] Rorbach J. , Gammage P.A., Minczuk M. C7orf30 is necessary for biogenesis of the large subunit of the mitochondrial ribosome. Nucleic Acids Res. 2012; 40:4097–4109. 22238376 10.1093/nar/gkr1282 PMC3351152

[26] Brown A. , Rathore S., Kimanius D., Aibara S., Bai X.C., Rorbach J., Amunts A., Ramakrishnan V. Structures of the human mitochondrial ribosome in native states of assembly. Nat. Struct. Mol. Biol. 2017; 24:866–869. 28892042 10.1038/nsmb.3464 PMC5633077

[27] Desai N. , Yang H., Chandrasekaran V., Kazi R., Minczuk M., Ramakrishnan V. Elongational stalling activates mitoribosome-associated quality control. Science. 2020; 370:1105–1110. 33243891 10.1126/science.abc7782 PMC7116630

[28] Bogenhagen D.F. , Martin D.W., Koller A. Initial steps in RNA processing and ribosome assembly occur at mitochondrial DNA nucleoids. Cell Metab. 2014; 19:618–629. 24703694 10.1016/j.cmet.2014.03.013

[29] Rackham O. , Busch J.D., Matic S., Siira S.J., Kuznetsova I., Atanassov I., Ermer J.A., Shearwood A.M., Richman T.R., Stewart J.B. et al. Hierarchical RNA processing is required for mitochondrial ribosome assembly. Cell Rep. 2016; 16:1874–1890. 27498866 10.1016/j.celrep.2016.07.031

[30] Dalla Rosa I. , Durigon R., Pearce S.F., Rorbach J., Hirst E.M., Vidoni S., Reyes A., Brea-Calvo G., Minczuk M., Woellhaf M.W. et al. MPV17L2 is required for ribosome assembly in mitochondria. Nucleic Acids Res. 2014; 42:8500–8515. 24948607 10.1093/nar/gku513 PMC4117752

[31] Bogenhagen D.F. , Ostermeyer-Fay A.G., Haley J.D., Garcia-Diaz M. Kinetics and mechanism of mammalian mitochondrial ribosome assembly. Cell Rep. 2018; 22:1935–1944. 29444443 10.1016/j.celrep.2018.01.066 PMC5855118

[32] Jourdain A.A. , Boehm E., Maundrell K., Martinou J.C. Mitochondrial RNA granules: compartmentalizing mitochondrial gene expression. J. Cell Biol. 2016; 212:611–614. 26953349 10.1083/jcb.201507125 PMC4792075

[33] Babu V.M.P. , Sankari S., Budnick J.A., Caswell C.C., Walker G.C Sinorhizobium meliloti YbeY is a zinc-dependent single-strand specific endoribonuclease that plays an important role in 16S ribosomal RNA processing. Nucleic Acids Res. 2020; 48:332–348. 31777930 10.1093/nar/gkz1095 PMC6943124

[34] Jacob A.I. , Kohrer C., Davies B.W., RajBhandary U.L., Walker G.C. Conserved bacterial RNase YbeY plays key roles in 70S ribosome quality control and 16S rRNA maturation. Mol. Cell. 2013; 49:427–438. 23273979 10.1016/j.molcel.2012.11.025 PMC3570609

[35] Smith B.A. , Gupta N., Denny K., Culver G.M. Characterization of 16S rRNA processing with pre-30S subunit assembly intermediates from *E. coli*. J. Mol. Biol. 2018; 430:1745–1759. 29660326 10.1016/j.jmb.2018.04.009

[36] Vercruysse M. , Kohrer C., Davies B.W., Arnold M.F., Mekalanos J.J., RajBhandary U.L., Walker G.C. The highly conserved bacterial RNase YbeY is essential in Vibrio cholerae, playing a critical role in virulence, stress regulation, and RNA processing. PLoS Pathog. 2014; 10:e1004175. 24901994 10.1371/journal.ppat.1004175 PMC4047096

[37] Rasouly A. , Davidovich C., Ron E.Z. The heat shock protein YbeY is required for optimal activity of the 30S ribosomal subunit. J. Bacteriol. 2010; 192:4592–4596. 20639334 10.1128/JB.00448-10 PMC2937419

[38] Rasouly A. , Schonbrun M., Shenhar Y., Ron E.Z. YbeY, a heat shock protein involved in translation in Escherichia coli. J. Bacteriol. 2009; 191:2649–2655. 19181801 10.1128/JB.01663-08 PMC2668401

[39] Baumgardt K. , Gilet L., Figaro S., Condon C. The essential nature of YqfG, a YbeY homologue required for 3′ maturation of *Bacillus subtilis* 16S ribosomal RNA is suppressed by deletion of RNase R. Nucleic Acids Res. 2018; 46:8605–8615. 29873764 10.1093/nar/gky488 PMC6144821

[40] Davies B.W. , Kohrer C., Jacob A.I., Simmons L.A., Zhu J., Aleman L.M., Rajbhandary U.L., Walker G.C. Role of *Escherichia coli* YbeY, a highly conserved protein, in rRNA processing. Mol. Microbiol. 2010; 78:506–518. 20807199 10.1111/j.1365-2958.2010.07351.x PMC2959132

[41] Dubin D.T. , Montoya J., Timko K.D., Attardi G. Sequence analysis and precise mapping of the 3′ ends of HeLa cell mitochondrial ribosomal RNAs. J. Mol. Biol. 1982; 157:1–19. 7108954 10.1016/0022-2836(82)90510-1

[42] Ghosal A. , Kohrer C., Babu V.M.P., Yamanaka K., Davies B.W., Jacob A.I., Ferullo D.J., Gruber C.C., Vercruysse M., Walker G.C C21orf57 is a human homologue of bacterial YbeY proteins. Biochem. Biophys. Res. Commun. 2017; 484:612–617. 28153719 10.1016/j.bbrc.2017.01.149 PMC5318257

[43] Di Nottia M. , Marchese M., Verrigni D., Mutti C.D., Torraco A., Oliva R., Fernandez-Vizarra E., Morani F., Trani G., Rizza T. et al. A homozygous MRPL24 mutation causes a complex movement disorder and affects the mitoribosome assembly. Neurobiol. Dis. 2020; 141:104880. 32344152 10.1016/j.nbd.2020.104880

[44] Garone C. , D'Souza A.R., Dallabona C., Lodi T., Rebelo-Guiomar P., Rorbach J., Donati M.A., Procopio E., Montomoli M., Guerrini R. et al. Defective mitochondrial rRNA methyltransferase MRM2 causes MELAS-like clinical syndrome. Hum. Mol. Genet. 2017; 26:4257–4266. 28973171 10.1093/hmg/ddx314 PMC5886288

[45] Nicholls T.J. , Rorbach J., Minczuk M. Mitochondria: mitochondrial RNA metabolism and human disease. Int. J. Biochem. Cell Biol. 2013; 45:845–849. 23333854 10.1016/j.biocel.2013.01.005

[46] Boczonadi V. , Ricci G., Horvath R. Mitochondrial DNA transcription and translation: clinical syndromes. Essays Biochem. 2018; 62:321–340. 29980628 10.1042/EBC20170103 PMC6056718

[47] Saramago M. , Peregrina A., Robledo M., Matos R.G., Hilker R., Serrania J., Becker A., Arraiano C.M., Jimenez-Zurdo J.I. Sinorhizobium meliloti YbeY is an endoribonuclease with unprecedented catalytic features, acting as silencing enzyme in riboregulation. Nucleic Acids Res. 2017; 45:1371–1391. 28180335 10.1093/nar/gkw1234 PMC5388416

[48] Liu J. , Zhou W., Liu G., Yang C., Sun Y., Wu W., Cao S., Wang C., Hai G., Wang Z. et al. The conserved endoribonuclease YbeY is required for chloroplast ribosomal RNA processing in Arabidopsis. Plant Physiol. 2015; 168:205–221. 25810095 10.1104/pp.114.255000 PMC4424013

[49] Minczuk M. , He J., Duch A.M., Ettema T.J., Chlebowski A., Dzionek K., Nijtmans L.G., Huynen M.A., Holt I.J. TEFM (c17orf42) is necessary for transcription of human mtDNA. Nucleic Acids Res. 2011; 39:4284–4299. 21278163 10.1093/nar/gkq1224 PMC3105396

[50] Bridges H.R. , Mohammed K., Harbour M.E., Hirst J. Subunit NDUFV3 is present in two distinct isoforms in mammalian complex I. Biochim. Biophys. Acta Bioenerg. 2017; 1858:197–207. 27940020 10.1016/j.bbabio.2016.12.001 PMC5293009

[51] Pearce S.F. , Rorbach J., Van Haute L., D'Souza A.R., Rebelo-Guiomar P., Powell C.A., Brierley I., Firth A.E., Minczuk M Maturation of selected human mitochondrial tRNAs requires deadenylation. Elife. 2017; 6:e27596. 28745585 10.7554/eLife.27596 PMC5544427

[52] Palenikova P. , Harbour M.E., Ding S., Fearnley I.M., Van Haute L., Rorbach J., Scavetta R., Minczuk M., Rebelo-Guiomar P. Quantitative density gradient analysis by mass spectrometry (qDGMS) and complexome profiling analysis (ComPrAn) R package for the study of macromolecular complexes. Biochim. Biophys. Acta Bioenerg. 2021; 1862:148399. 33592209 10.1016/j.bbabio.2021.148399 PMC8047798

[53] Heide H. , Bleier L., Steger M., Ackermann J., Drose S., Schwamb B., Zornig M., Reichert A.S., Koch I., Wittig I. et al. Complexome profiling identifies TMEM126B as a component of the mitochondrial complex I assembly complex. Cell Metab. 2012; 16:538–549. 22982022 10.1016/j.cmet.2012.08.009

[54] Protasoni M. , Perez-Perez R., Lobo-Jarne T., Harbour M.E., Ding S., Penas A., Diaz F., Moraes C.T., Fearnley I.M., Zeviani M. et al. Respiratory supercomplexes act as a platform for complex III-mediated maturation of human mitochondrial complexes I and IV. EMBO J. 2020; 39:e102817. 31912925 10.15252/embj.2019102817 PMC6996572

[55] Palenikova P. , Harbour M.E., Prodi F., Minczuk M., Zeviani M., Ghelli A., Fernandez-Vizarra E. Duplexing complexome profiling with SILAC to study human respiratory chain assembly defects. Biochim. Biophys. Acta Bioenerg. 2021; 1862:148395. 33600785 10.1016/j.bbabio.2021.148395

[56] Penhoat C.H. , Li Z., Atreya H.S., Kim S., Yee A., Xiao R., Murray D., Arrowsmith C.H., Szyperski T. NMR solution structure of *Thermotoga maritima* protein TM1509 reveals a Zn-metalloprotease-like tertiary structure. J. Struct. Funct. Genomics. 2005; 6:51–62. 15965736 10.1007/s10969-005-5277-z

[57] Calvo S.E. , Clauser K.R., Mootha V.K. MitoCarta2.0: an updated inventory of mammalian mitochondrial proteins. Nucleic Acids Res. 2016; 44:D1251–D1257. 26450961 10.1093/nar/gkv1003 PMC4702768

[58] Smith A.C. , Robinson A.J. MitoMiner v3.1, an update on the mitochondrial proteomics database. Nucleic Acids Res. 2016; 44:D1258–D1261. 26432830 10.1093/nar/gkv1001 PMC4702766

[59] Arroyo J.D. , Jourdain A.A., Calvo S.E., Ballarano C.A., Doench J.G., Root D.E., Mootha V.K. A Genome-wide CRISPR death screen identifies genes essential for oxidative phosphorylation. Cell Metab. 2016; 24:875–885. 27667664 10.1016/j.cmet.2016.08.017 PMC5474757

[60] Small I. , Peeters N., Legeai F., Lurin C. Predotar: A tool for rapidly screening proteomes for N-terminal targeting sequences. Proteomics. 2004; 4:1581–1590. 15174128 10.1002/pmic.200300776

[61] Emanuelsson O. , Nielsen H., Brunak S., von Heijne G. Predicting subcellular localization of proteins based on their N-terminal amino acid sequence. J. Mol. Biol. 2000; 300:1005–1016. 10891285 10.1006/jmbi.2000.3903

[62] Claros M.G. , Vincens P. Computational method to predict mitochondrially imported proteins and their targeting sequences. Eur. J. Biochem. 1996; 241:779–786. 8944766 10.1111/j.1432-1033.1996.00779.x

[63] Antonicka H. , Sasarman F., Nishimura T., Paupe V., Shoubridge E.A. The mitochondrial RNA-binding protein GRSF1 localizes to RNA granules and is required for posttranscriptional mitochondrial gene expression. Cell Metab. 2013; 17:386–398. 23473033 10.1016/j.cmet.2013.02.006

[64] Metodiev M.D. , Lesko N., Park C.B., Camara Y., Shi Y., Wibom R., Hultenby K., Gustafsson C.M., Larsson N.G. Methylation of 12S rRNA is necessary for in vivo stability of the small subunit of the mammalian mitochondrial ribosome. Cell Metab. 2009; 9:386–397. 19356719 10.1016/j.cmet.2009.03.001

[65] Rorbach J. , Nicholls T.J., Minczuk M. PDE12 removes mitochondrial RNA poly(A) tails and controls translation in human mitochondria. Nucleic Acids Res. 2011; 39:7750–7763. 21666256 10.1093/nar/gkr470 PMC3177208

[66] Wydro M. , Bobrowicz A., Temperley R.J., Lightowlers R.N., Chrzanowska-Lightowlers Z.M. Targeting of the cytosolic poly(A) binding protein PABPC1 to mitochondria causes mitochondrial translation inhibition. Nucleic Acids Res. 2010; 38:3732–3742. 20144953 10.1093/nar/gkq068 PMC2887948

[67] Vercruysse M. , Kohrer C., Shen Y., Proulx S., Ghosal A., Davies B.W., RajBhandary U.L., Walker G.C. Identification of YbeY-protein interactions involved in 16S rRNA maturation and stress regulation in escherichia coli. MBio. 2016; 7:e01785-16. 10.1128/mBio.01785-16 PMC5101352 27834201

[68] D'Souza A.R. , Minczuk M. Mitochondrial transcription and translation: overview. Essays Biochem. 2018; 62:309–320. 30030363 10.1042/EBC20170102 PMC6056719

[69] Rossmanith W. Processing of human mitochondrial tRNA(Ser(AGY))GCU: a novel pathway in tRNA biosynthesis. J. Mol. Biol. 1997; 265:365–371. 9034356 10.1006/jmbi.1996.0750

[70] Haack T.B. , Kopajtich R., Freisinger P., Wieland T., Rorbach J., Nicholls T.J., Baruffini E., Walther A., Danhauser K., Zimmermann F.A. et al. ELAC2 mutations cause a mitochondrial RNA processing defect associated with hypertrophic cardiomyopathy. Am. J. Hum. Genet. 2013; 93:211–223. 23849775 10.1016/j.ajhg.2013.06.006 PMC3738821

[71] Summer S. , Smirnova A., Gabriele A., Toth U., Fasemore A.M., Forstner K.U., Kuhn L., Chicher J., Hammann P., Mitulovic G. et al. YBEY is an essential biogenesis factor for mitochondrial ribosomes. Nucleic Acids Res. 2020; 48:9762–9786. 32182356 10.1093/nar/gkaa148 PMC7515705

[72] Szczepanowska K. , Maiti P., Kukat A., Hofsetz E., Nolte H., Senft K., Becker C., Ruzzenente B., Hornig-Do H.T., Wibom R. et al. CLPP coordinates mitoribosomal assembly through the regulation of ERAL1 levels. EMBO J. 2016; 35:2566–2583. 27797820 10.15252/embj.201694253 PMC5283601

[73] Chen S.S. , Sperling E., Silverman J.M., Davis J.H., Williamson J.R. Measuring the dynamics of E. coli ribosome biogenesis using pulse-labeling and quantitative mass spectrometry. Mol. Biosyst. 2012; 8:3325–3334. 23090316 10.1039/c2mb25310k PMC3501348

[74] Loughran G. , Zhdanov A.V., Mikhaylova M.S., Rozov F.N., Datskevich P.N., Kovalchuk S.I., Serebryakova M.V., Kiniry S.J., Michel A.M., O’Connor P.B.F. et al. Unusually efficient CUG initiation of an overlapping reading frame in POLG mRNA yields novel protein POLGARF. Proc. Natl. Acad. Sci. U.S.A. 2020; 117:24936–24946. 32958672 10.1073/pnas.2001433117 PMC7547235

[75] Su W.P. , Wang W.J., Chang J.Y., Ho P.C., Liu T.Y., Wen K.Y., Kuo H.L., Chen Y.J., Huang S.S., Subhan D. et al. Therapeutic Zfra4-10 or WWOX7-21 peptide induces complex formation of WWOX with selective protein targets in organs that leads to cancer suppression and spleen cytotoxic memory Z Cell activation in vivo. Cancers (Basel). 2020; 12:2189. 10.3390/cancers12082189 PMC7464583 32764489

[76] Feichtinger R.G. , Olahova M., Kishita Y., Garone C., Kremer L.S., Yagi M., Uchiumi T., Jourdain A.A., Thompson K., D'Souza A.R. et al. Biallelic C1QBP mutations cause severe neonatal-, Childhood-, or later-onset cardiomyopathy associated with combined respiratory-chain deficiencies. Am. J. Hum. Genet. 2017; 101:525–538. 28942965 10.1016/j.ajhg.2017.08.015 PMC5630164

[77] Ghate N.B. , Kim J., Shin Y., Situ A., Ulmer T.S., An W. p32 is a negative regulator of p53 tetramerization and transactivation. Mol Oncol. 2019; 13:1976–1992. 31293051 10.1002/1878-0261.12543 PMC6717765

[78] Matthews D.A. , Russell W.C. Adenovirus core protein V interacts with p32–a protein which is associated with both the mitochondria and the nucleus. J. Gen. Virol. 1998; 79:1677–1685. 9680131 10.1099/0022-1317-79-7-1677

[79] Itahana K. , Zhang Y. Mitochondrial p32 is a critical mediator of ARF-induced apoptosis. Cancer Cell. 2008; 13:542–553. 18538737 10.1016/j.ccr.2008.04.002 PMC4504427

[80] Jiao H. , Su G.Q., Dong W., Zhang L., Xie W., Yao L.M., Chen P., Wang Z.X., Liou Y.C., You H. Chaperone-like protein p32 regulates ULK1 stability and autophagy. Cell Death Differ. 2015; 22:1812–1823. 25909887 10.1038/cdd.2015.34 PMC4648329

[81] Yagi M. , Uchiumi T., Takazaki S., Okuno B., Nomura M., Yoshida S., Kanki T., Kang D p32/gC1qR is indispensable for fetal development and mitochondrial translation: importance of its RNA-binding ability. Nucleic Acids Res. 2012; 40:9717–9737. 22904065 10.1093/nar/gks774 PMC3479211

[82] Murgia M. , Tan J., Geyer P.E., Doll S., Mann M., Klopstock T. Proteomics of cytochrome c oxidase-negative versus -positive muscle fiber sections in mitochondrial myopathy. Cell Rep. 2019; 29:3825–3834. 31851916 10.1016/j.celrep.2019.11.055

[83] Metodiev M.D. , Spahr H., Loguercio Polosa P., Meharg C., Becker C., Altmueller J., Habermann B., Larsson N.G., Ruzzenente B. NSUN4 is a dual function mitochondrial protein required for both methylation of 12S rRNA and coordination of mitoribosomal assembly. PLos Genet. 2014; 10:e1004110. 24516400 10.1371/journal.pgen.1004110 PMC3916286

[84] Spahr H. , Habermann B., Gustafsson C.M., Larsson N.G., Hallberg B.M. Structure of the human MTERF4-NSUN4 protein complex that regulates mitochondrial ribosome biogenesis. Proc. Natl. Acad. Sci. U.S.A. 2012; 109:15253–15258. 22949673 10.1073/pnas.1210688109 PMC3458362

[85] Vilardo E. , Nachbagauer C., Buzet A., Taschner A., Holzmann J., Rossmanith W. A subcomplex of human mitochondrial RNase P is a bifunctional methyltransferase–extensive moonlighting in mitochondrial tRNA biogenesis. Nucleic Acids Res. 2012; 40:11583–11593. 23042678 10.1093/nar/gks910 PMC3526285

