# Supplementary material for: YbeY is required for ribosome small subunit assembly and tRNA processing in human mitochondria
Source: Nucleic Acids Res. 2021 May 25;49(10):5798–812. doi: 10.1093/nar/gkab404 (PMC8191802; doi:10.1093/nar/gkab404)
Supplement: gkab404_Supplemental_Files [file gkab404_supplemental_files.zip › YbeY R3 Suppl. Materials MAM200421.docx]

**Supplementary Information**

**YbeY is required for ribosome small subunit assembly and tRNA processing in human mitochondria**

Aaron R. D’Souza^1^, Lindsey Van Haute^1^, Christopher A. Powell^1^, Christian Daniel Mutti^1^, Petra Páleníková^1^, Pedro Rebelo-Guiomar^1^, Joanna Rorbach^1,2^, Michal Minczuk^1,4^

^1^ Medical Research Council Mitochondrial Biology Unit, University of Cambridge, Hills Road, Cambridge, CB2 0XY, UK

^2^ Present address: Department of Medical Biochemistry and Biophysics, Karolinska Institutet, Biomedicum, Solnavägen 9, 171 65 Solna


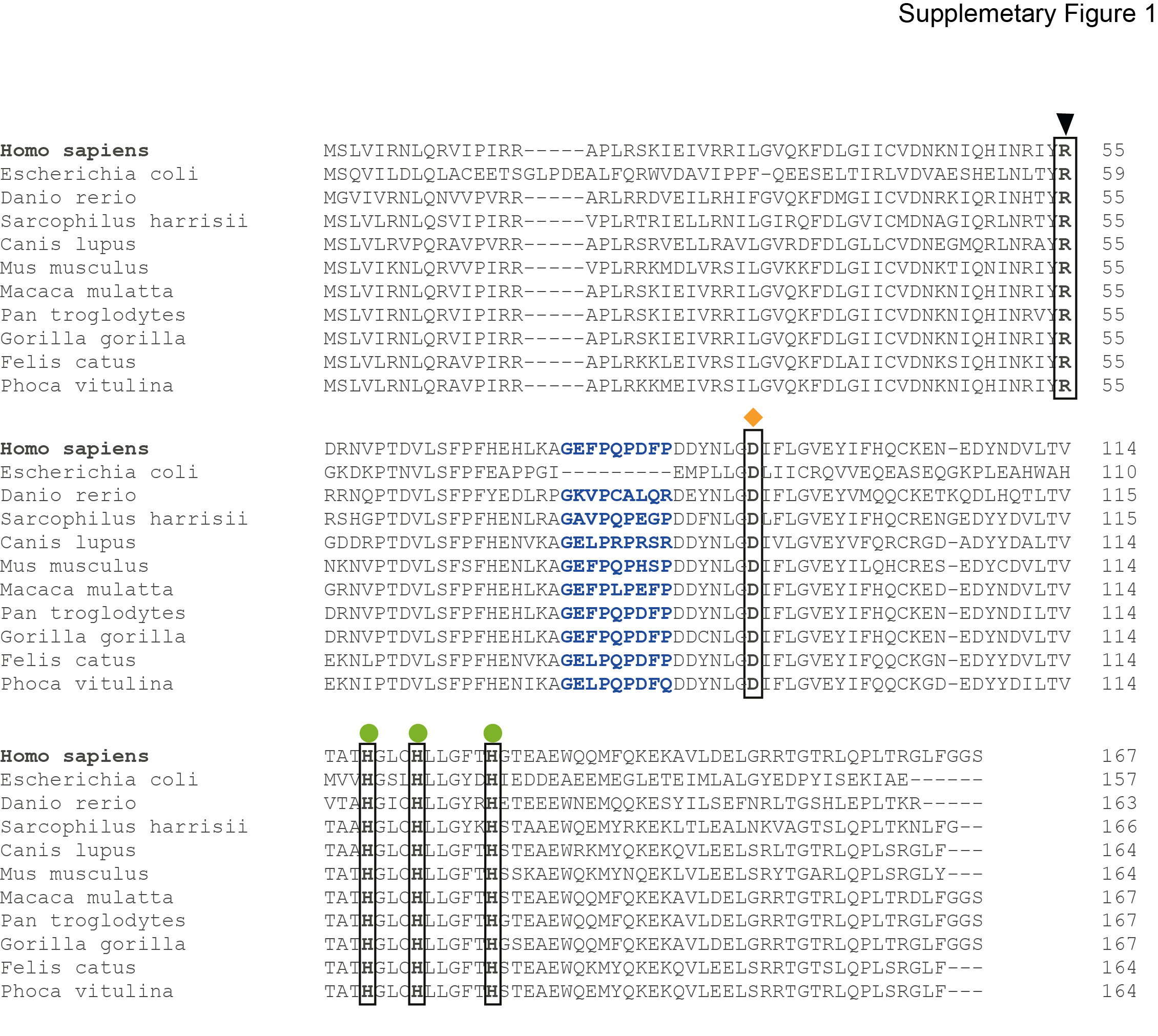


**Supplementary Figure S1 (related to Figure 1)** | **Sequence conservation in YbeY**

The YbeY amino acid sequences of various species were aligned using Clustal Omega tool. The catalytically-important RNA-binding arginine is indicated by a black triangle above. Green circles indicate histidine residues from the zinc ion-binding H3H5XH motif. Asp90 (equivalent of Asp85 in
*E. coli*), found in the beta-sheet outside the active site and required for the interaction of YbeY with the ribosomal SSU component S11 is indicated with an orange diamond. Eukaryote-specific insertion is indicated in blue.

**
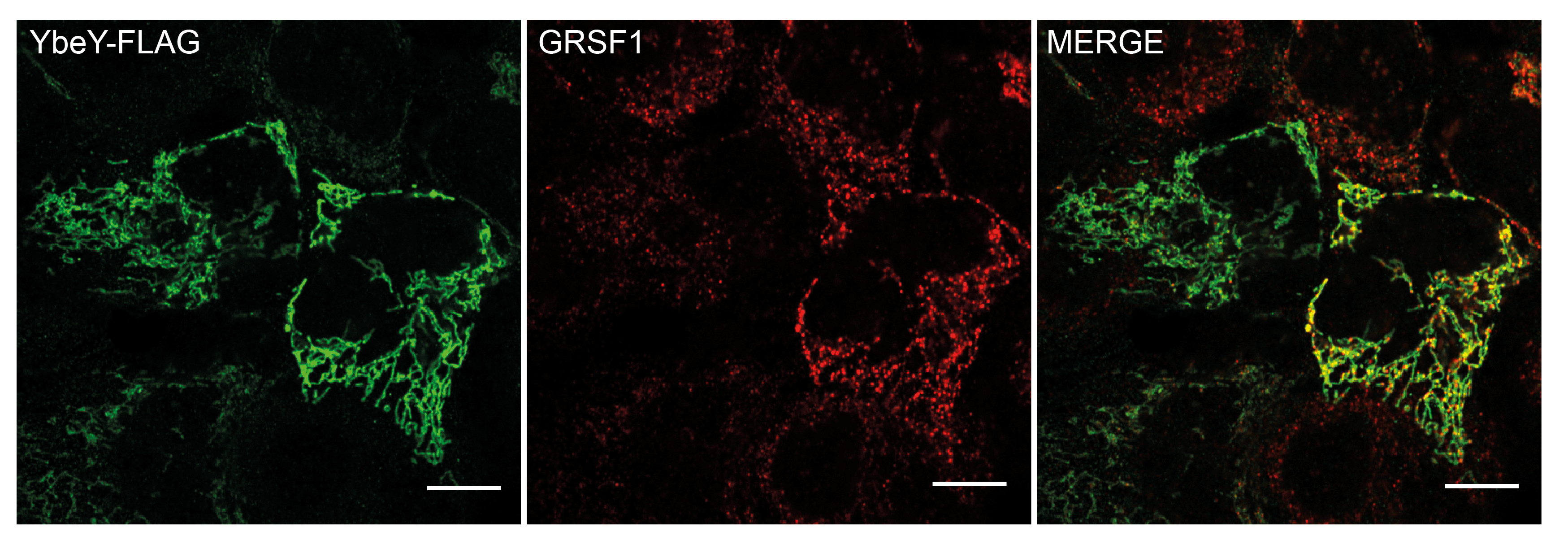
**

**Supplementary Figure S2 (related to Figure 1)** | **Investigating YbeY colocalization in mitochondrial RNA granules using immunocytochemistry.**

Flag-tagged YbeY was transiently expressed in HeLa cells and visualised using anti-flag antibodies and visualised using secondary antibodies conjugated with Alexa fluor 488 (green, left). RNA granules were stained using antibodies against GRSF1 (red, middle). Co-localisation is shown in yellow. The white scale bar at the bottom depicts 10 µm.


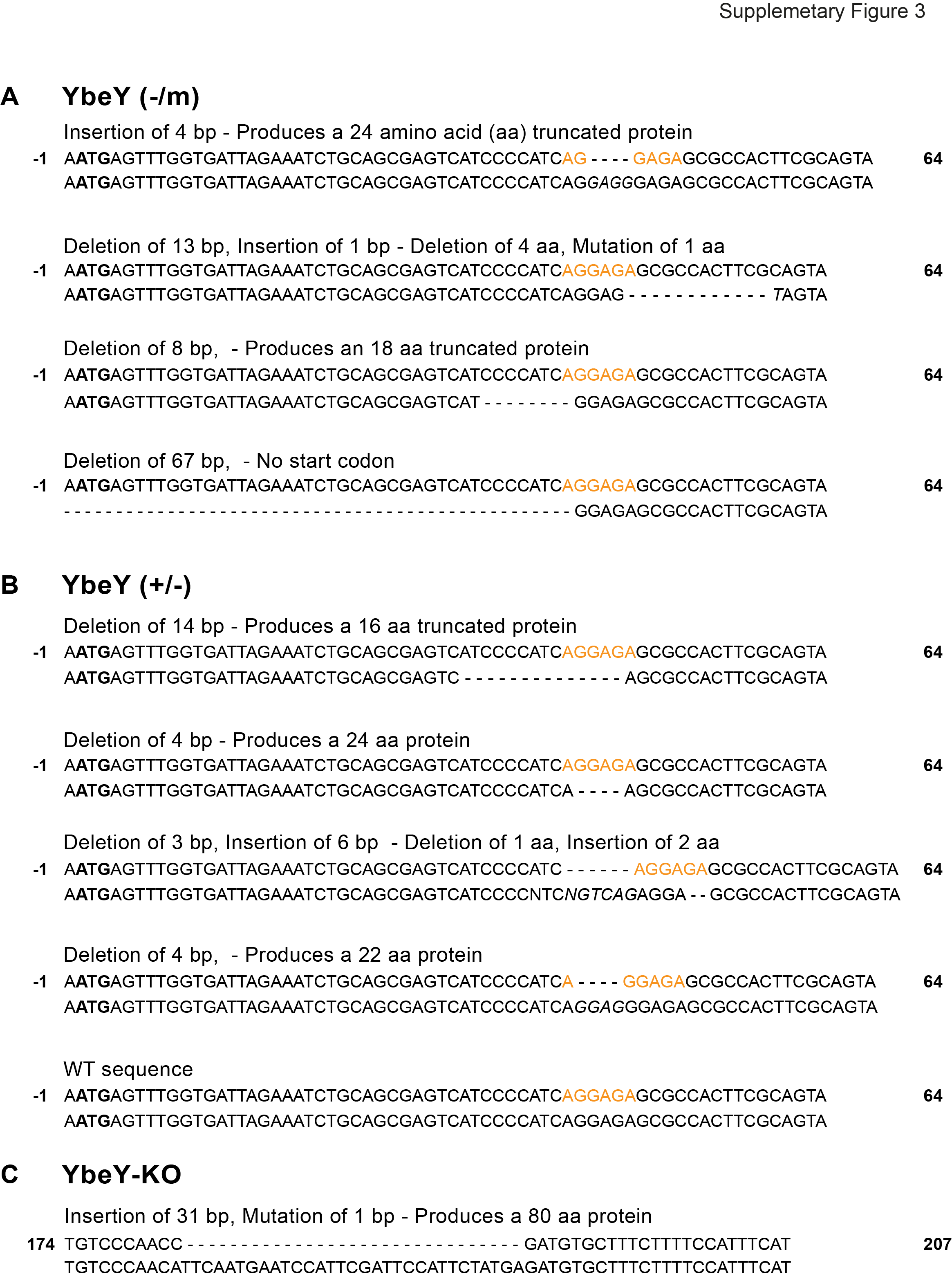


**Supplementary figure S3 (related to Figure 2)** | **Genome modifications of the *YbeY* locus.**

PCR products from candidates were cloned into pCR4 vectors using Zero Blunt TOPO cloning kit and bacterial colonies were picked and analysed by Sanger sequencing. The full-length protein is 167 amino acids (aa) long. Indels at the ZFN target sites are shown. **(A)** Four alleles of YbeY were identified in the YbeY(-/m) cell line **(B)** Five alleles of YbeY were identified in the YbeY(+/-) line. The wild type sequence is above in the pair of sequences. NB: one of the alleles is WT. The Start codon is in bold. The zinc finger nuclease target site is coloured orange. **(C)** A single allele is present in the YbeY-KO Hap1 cells.

**
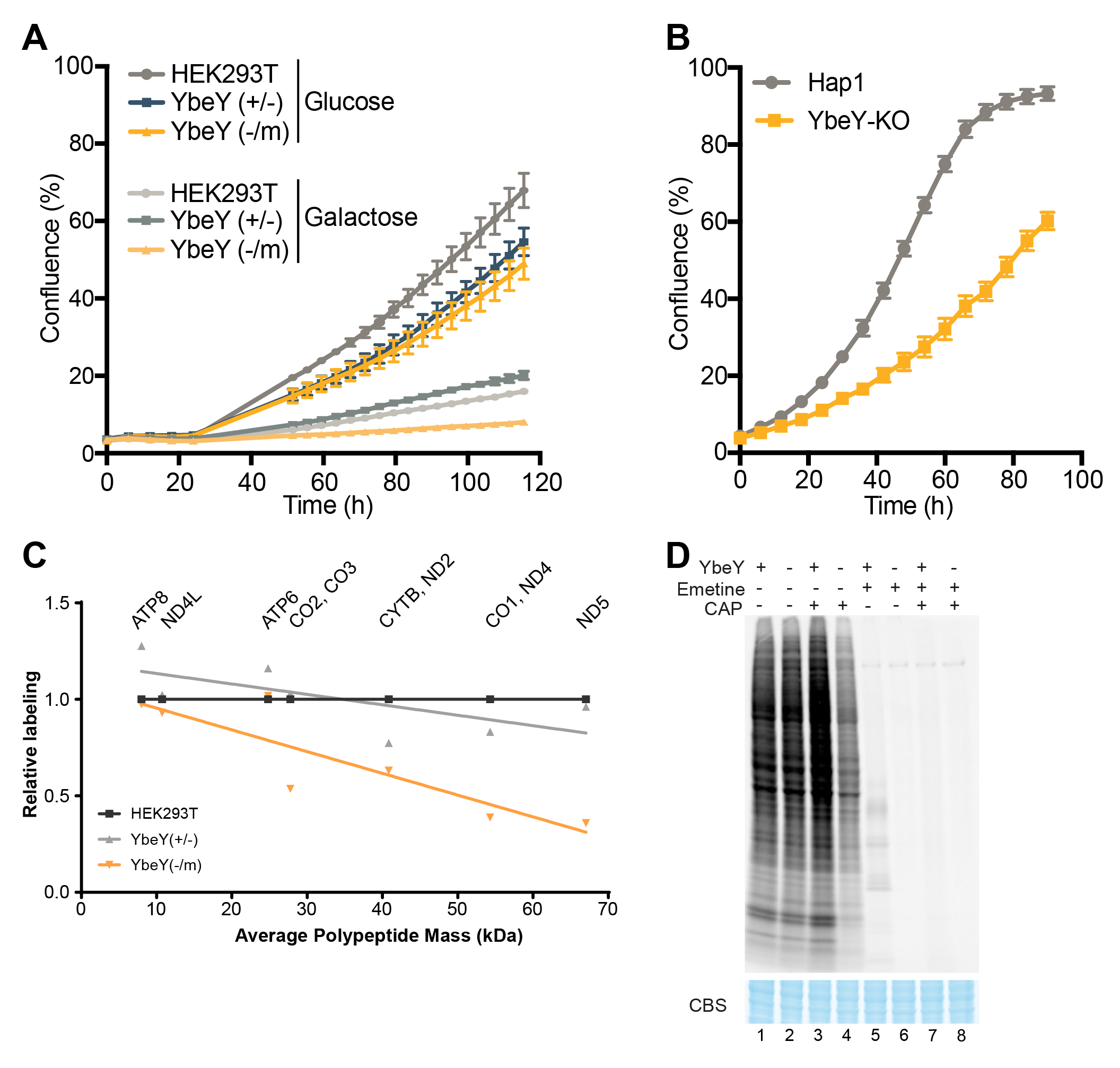
**

**Supplementary Figure 4 (related to Figure 2) | Mitochondrial translation in YbeY-deficient cells.**

**(A)** Growth of HEK293T parental and YbeY-deficient HEK293T cells in glucose and galactose media. Representative experiment is shown where cells were plated in triplicate (mean ±1 SD). **(B)** Growth of Hap1 parental and YbeY knockout Hap1 cells (YbeY-KO) in high glucose IMDM media containing 20% FBS. Representative experiment is shown where cells were plated in triplicate (mean ±1 SD). **(C)** Quantification of mitochondrial translation products relative to the expected size of the polypeptide. Relative metabolic labelling of the polypeptides was quantified from the intensity of the bands (in Figure 2C) using ImageJ, measured relative to the control wild type sample. The bands specific for CO2 and CO3, CYTB and ND2, CO1 and ND4 were quantified together due to their proximity on the gel. The average mass of both proteins was calculated. The line represents the linear regression. YbeY(+/-) R^2^=0.46, YbeY(-/m) R^2^=0.77. **(D)** Metabolic labelling of translation products in Hap1 parental cells and YbeY knockout Hap1 cells in the presence and absence of cytoplasmic inhibitor, emetine, and mitochondrial inhibitor, chloramphenicol. Lanes 5 and 6 are identical to the experiment shown in Fig. 2F. Coomassie Blue staining (CBS) was used as loading control.


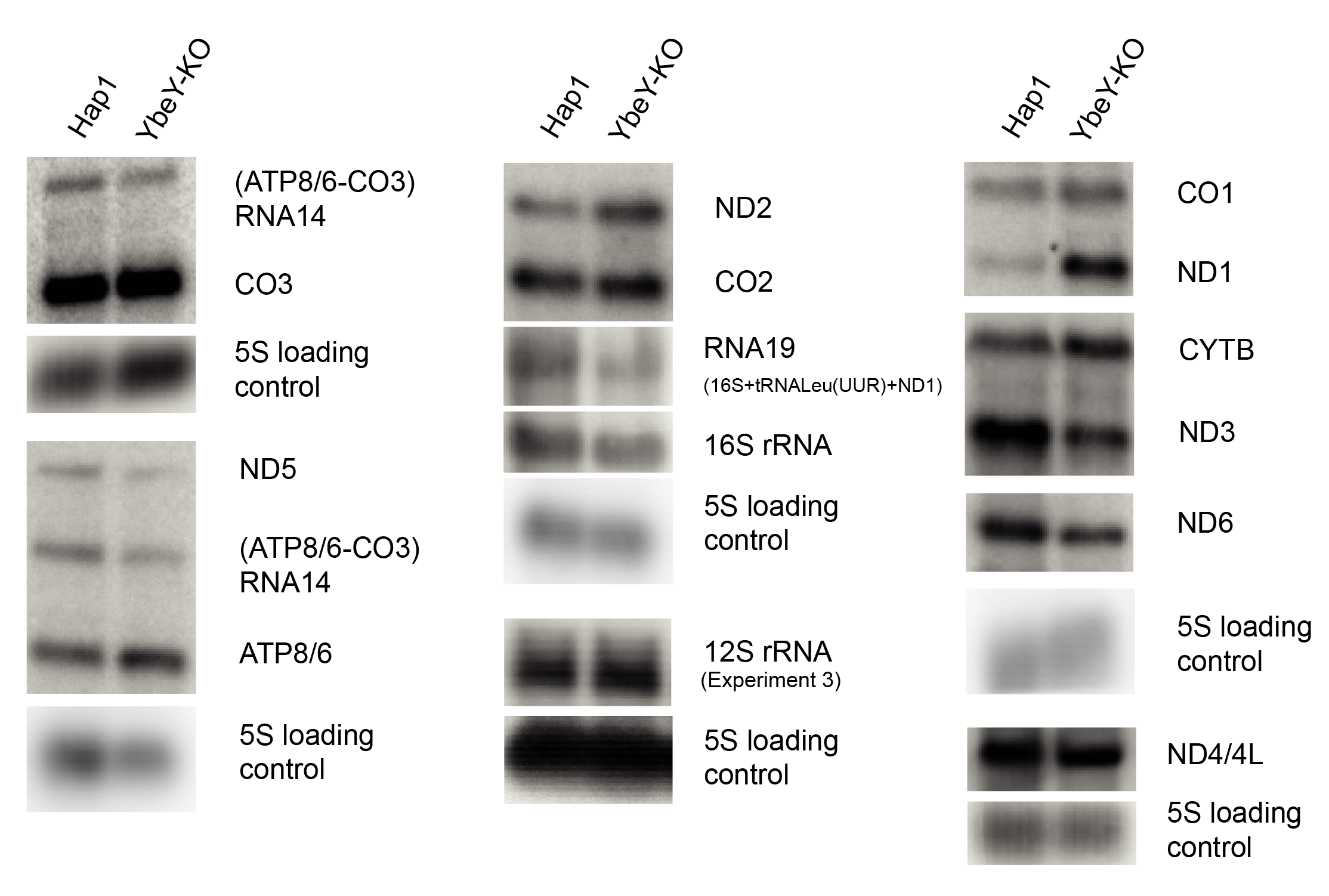


**Supplementary Figure 5 (related to Figure 3) | Northern blot analysis of mitochondrial transcripts.**

Northern blot of mitochondrial mRNAs in YbeY knockout Hap1 cells grown in standard IMDM medium**.** 5S rRNA was used as a loading control.


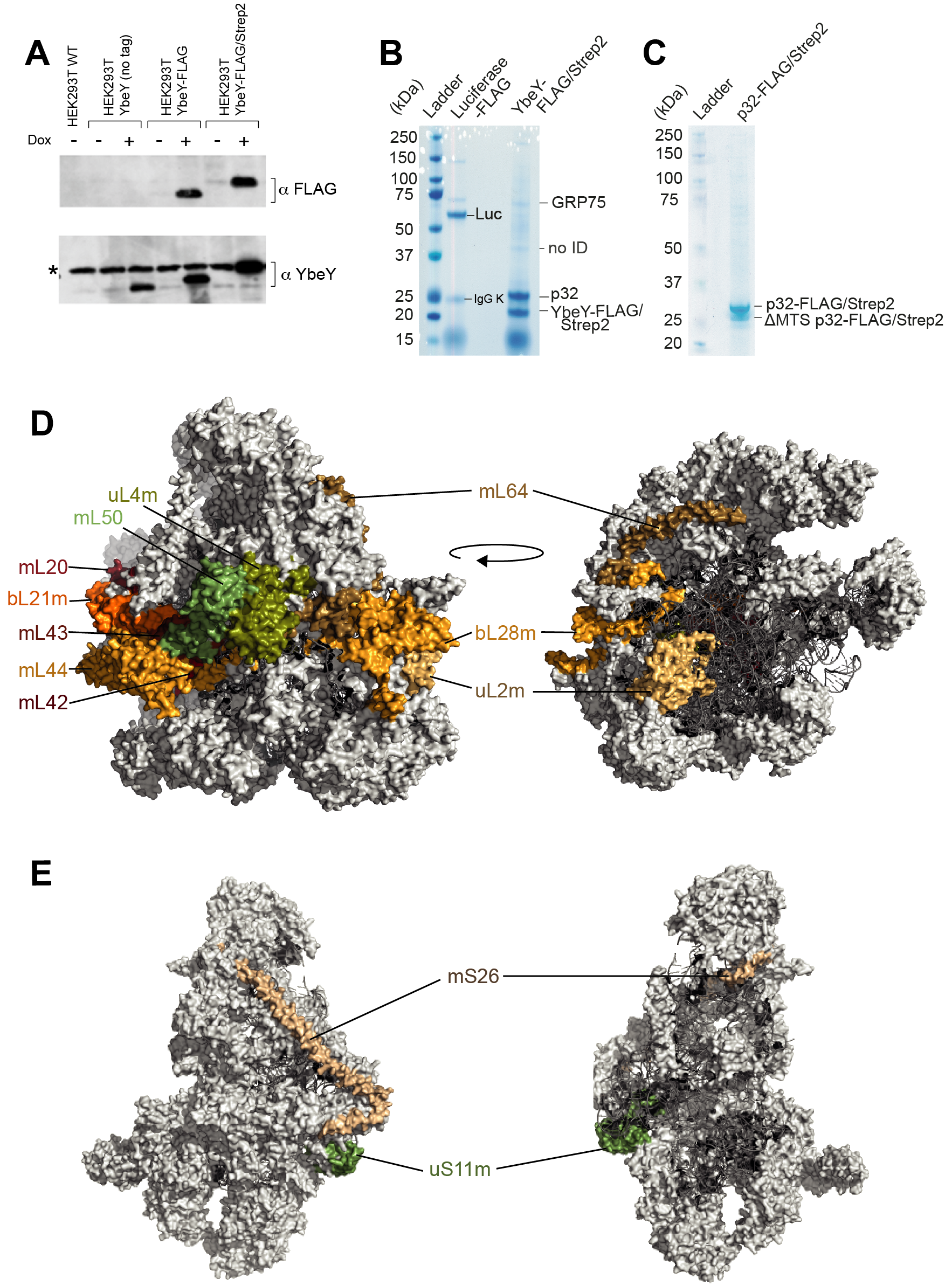


**Supplementary Figure S6 (related to Figure 5) |** **The analysis of YbeY interactors**.

**(A)** The YbeY variants integrated into the Frt site of the HEK293T/Flp-In/Trex cells were induced using doxycycline (Dox “+”). Uninduced cells (“-“) and HEK293T/Flp-In/Trex parental cells (WT) were used as controls. The cell lysates were run on a 10-20% Tris-glycine SDS PAGE gel. The proteins were transferred onto a nitrocellulose membrane and probed with the anti-FLAG antibody to identify the proteins with a FLAG tag or FLAG/Strep2 tag or the anti-YbeY antibody. * denotes unspecific protein. **(B)**-**(C)** Immunoaffinity pull-down of YbeY and p32/C1QBP. Proteins containing a C-terminal FLAG/Strep2-tag were overexpressed in the HEK293T/Flp-In/Trex cell lines using 100 ng/ml doxycycline, mitochondria were isolated from the cells, the mitochondrial lysate was incubated with the agarose beads coated with antibodies against the FLAG epitope and the bound proteins were eluted using flag peptide. The eluent was resolved on a 4-12% Bis-Tris SDS-PAGE gel and prominent bands were cut for analysis using LC-MS/MS (B) Pull-down of FLAG/Strep2-tagged YbeY. FLAG-tagged Luciferase (Luc) was used as a control. (C) Pull-down of FLAG/Strep2-tagged P32/C1QBP. ∆MTS p32-FLAG/Strep2 – The tagged p32/C1qBP with mitochondrial targeting sequence cleaved off. **(D)**-**(E)** Mitoribosomal proteins (light grey), rRNA (dark grey). (C) mt-LSU (D) mt-SSU (PDB: 3J9M). The bL20m-bL21m-mL42-mL43-mL44 subcomplex (red and orange labels) and uL4m-mL50 subcomplex (green labels).


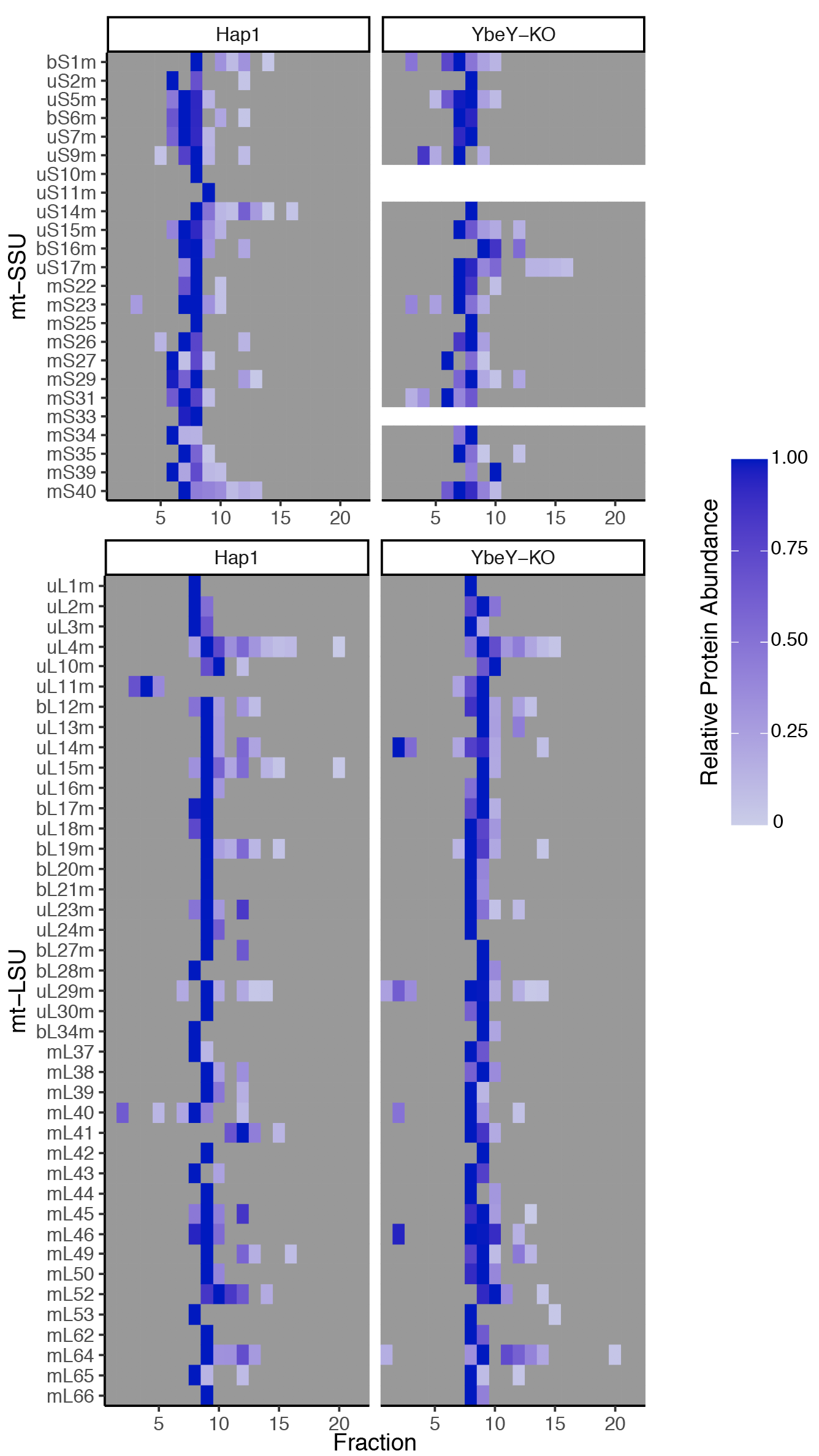


**Supplementary Figure S7 (related to Figure 6) |** **Qualitative comparison of mt-SSU and mt-LSU proteins in Hap1 WT and YbeY knock-out cells.**

Protein distribution in the fractions analysed by the qDGMS/ComPrAn pipeline. The heavy and light labelling states were analysed independently i.e. peptides were split into two groups according to their attributed labelling states. The peptide present in the highest number of fractions within each labelling state was considered the representative peptide. Thus, for the same protein, the peptide picked for one labelling state may be different from the peptide selected for the other. This scenario picks a representative peptide for all proteins, even if they are present only in one labelling state. Grey squares represent the absence of detection of a given protein in a given fraction. When a protein was not detected in any fraction a white line is shown. Mitoribosomal proteins not detected in WT cells are not shown.

**
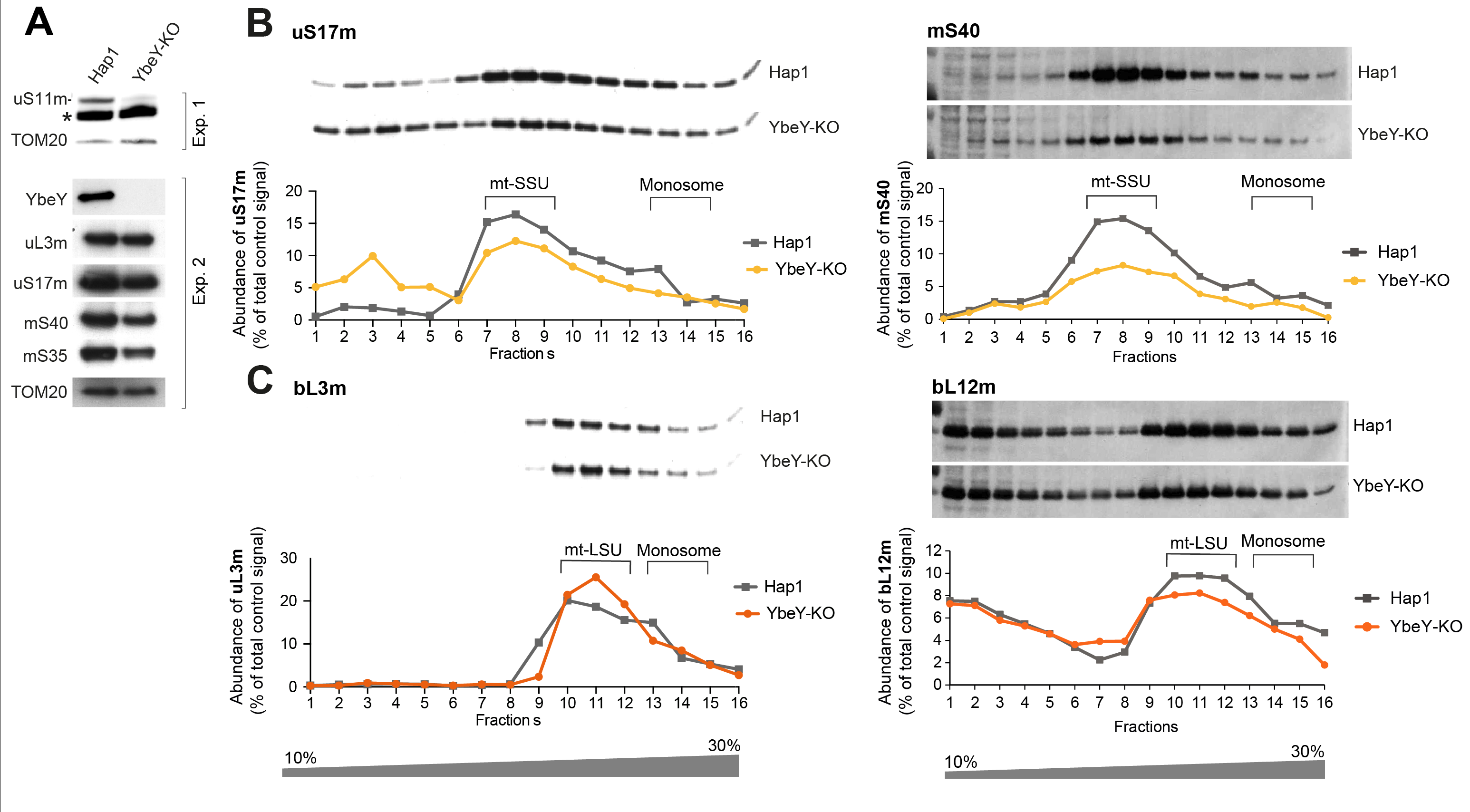
**

**Supplementary Figure S8 (related to Figure 6) |** **Mitochondrial SSU assembly is reduced in YbeY knockout Hap1 cells**

**(A)** Analysis of steady state levels of mitoribosomal proteins and endogenous YbeY in Hap1 parental and YbeY knockout Hap1 (YbeY-KO) cells by western blot. Two experiments are shown. TOM22 was used as a loading control. Asterisk indicates an unspecific band. **(B)** Analysis of the integrity of mt-SSU (uS17m, mS40) using isokinetic sucrose gradient fractionation and western blotting in YbeY-KO and Hap1 WT cells. **(C)** Analysis of the integrity of mt-LSU (uL3m, bL12m) using isokinetic sucrose gradient fractionation and western blotting in YbeY-KO and Hap1 WT cells.


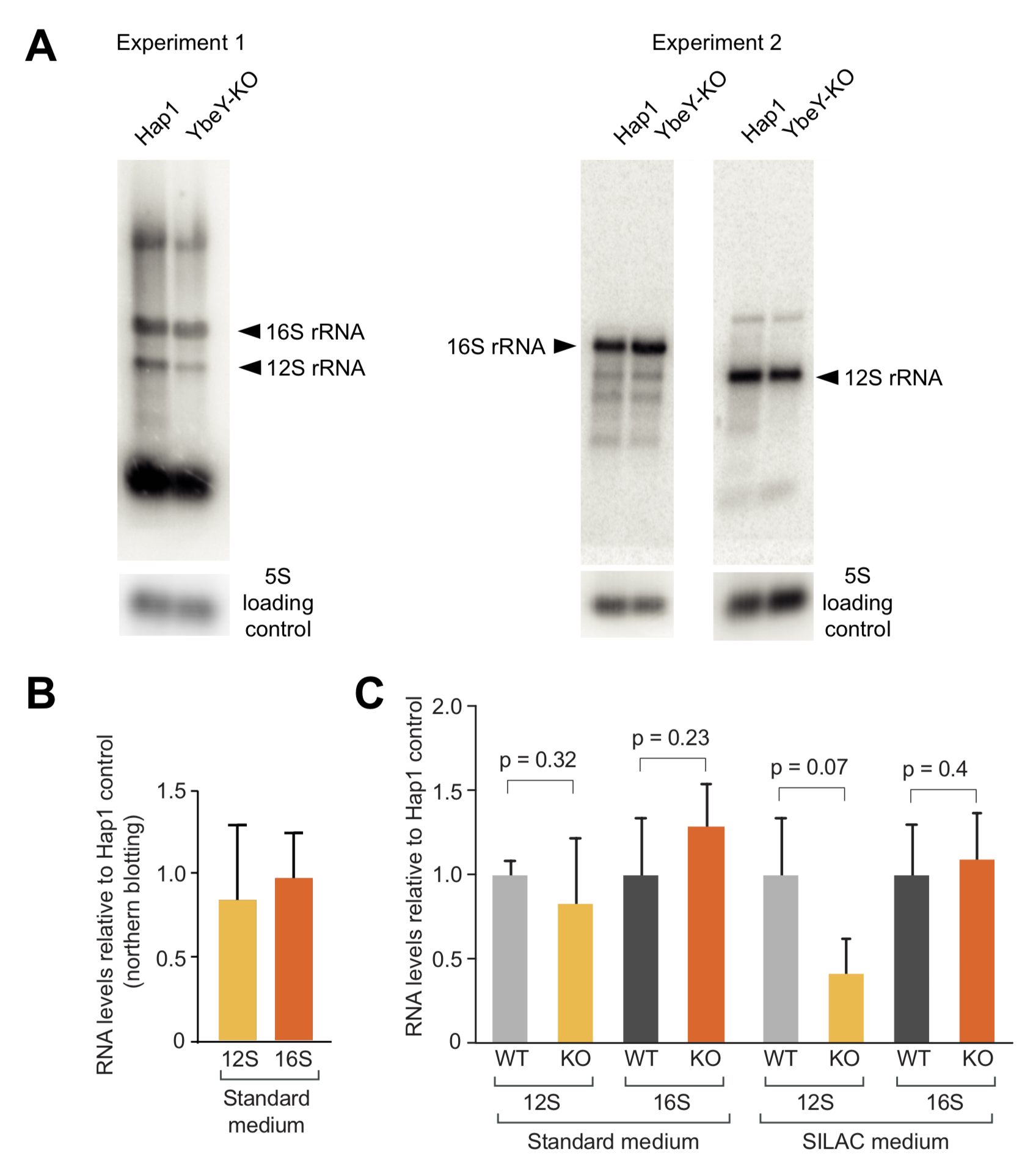


**Supplementary Figure S9 (related to Figure 6) |** **Steady state levels of mitochondrial rRNA in YbeY depleted cells in standard and SILAC medium**

**(A)** Uncropped northern blot experiments showing 12S rRNA and 16S rRNA in YbeY knockout Hap1 cells grown in standard IMDM medium indicating no accumulation of precursor rRNAs. 5S rRNA was used as a loading control. **(B)** Steady-state levels of 12S rRNA and 16S rRNA quantified by northern blotting in Hap1 control and YbeY knockout Hap1 cells grown in standard IMDM medium**.** 5S RNA was used as a loading control. Quantification of steady state levels was performed using Image J and normalised to the 5S RNA loading control and values from Hap1 control cells. Error bars = 1 SD.
**(C)** Comparison of 12S and 16S transcripts levels in Hap1 YbeY KO cells grown in regular IMDM medium supplemented with a 10% non-dialyzed FBS (Standard medium) or in IMDM medium for SILAC (deficient in L-lysine and L-arginine) supplemented with L-proline (200 μg/ml), L-lysine (146 μg/ml), L-arginine (69 μg/ml) and 10% dialyzed FBS (SILAC medium). Steady-state levels of 12S and 16S mt-rRNA were determined by RT-qPCR, normalized to GAPDH and are presented as values relative to Hap1 wild-type control cells. n=3. Error bars = SEM. Statistical analysis was carried out using a two-tailed Student’s t-test.
